# Mammalian ALKBH1 serves as an *N*^6^-mA demethylase of unpairing DNA

**DOI:** 10.1038/s41422-019-0237-5

**Published:** 2020-02-12

**Authors:** Min Zhang, Shumin Yang, Raman Nelakanti, Wentao Zhao, Gaochao Liu, Zheng Li, Xiaohui Liu, Tao Wu, Andrew Xiao, Haitao Li

**Affiliations:** 1 MOE Key Laboratory of Protein Sciences, Beijing Advanced Innovation Center for Structural Biology, Beijing Frontier Research Center for Biological Structure, Tsinghua-Peking Joint Center for Life Sciences, Department of Basic Medical Sciences, School of Medicine, Tsinghua University, 100084 Beijing, China; 2 Department of Genetics and Yale Stem Cell Center, Yale School of Medicine, New Haven, CT 06520 USA; 3 National Protein Science Technology Center, School of Life Sciences, Tsinghua University, 100084 Beijing, China; 4 Present Address: Department of Molecular and Human Genetics, Baylor College of Medicine, Houston, TX 77030 USA

**Keywords:** Methylation, DNA methylation, Epigenetic memory, X-ray crystallography

## Abstract

*N*^6^-methyladenine (*N*^6^-mA) of DNA is an emerging epigenetic mark in mammalian genome. Levels of *N*^6^-mA undergo drastic fluctuation during early embryogenesis, indicative of active regulation. Here we show that the 2-oxoglutarate-dependent oxygenase ALKBH1 functions as a nuclear eraser of *N*^6^-mA in unpairing regions (e.g., SIDD, Stress-Induced DNA Double Helix Destabilization regions) of mammalian genomes. Enzymatic profiling studies revealed that ALKBH1 prefers bubbled or bulged DNAs as substrate, instead of single-stranded (ss-) or double-stranded (ds-) DNAs. Structural studies of ALKBH1 revealed an unexpected “stretch-out” conformation of its “Flip1” motif, a conserved element that usually bends over catalytic center to facilitate substrate base flipping in other DNA demethylases. Thus, lack of a bending “Flip1” explains the observed preference of ALKBH1 for unpairing substrates, in which the flipped *N*^6^-mA is primed for catalysis. Co-crystal structural studies of ALKBH1 bound to a 21-mer bulged DNA explained the need of both flanking duplexes and a flipped base for recognition and catalysis. Key elements (e.g., an ALKBH1-specific α1 helix) as well as residues contributing to structural integrity and catalytic activity were validated by structure-based mutagenesis studies. Furthermore, ssDNA-seq and DIP-seq analyses revealed significant co-occurrence of base unpairing regions with *N*^6^-mA in mouse genome. Collectively, our biochemical, structural and genomic studies suggest that ALKBH1 is an important DNA demethylase that regulates genome *N*^6^-mA turnover of unpairing regions associated with dynamic chromosome regulation.

## Introduction

AlkB family of 2-oxoglutarate (2OG or α-KG)-dependent oxygenases are evolutionarily conserved and implicated in nucleotide demethylation.^1–3^ As a founding member of its family, AlkB homolog 1 (ALKBH1) was firstly identified in 1996;^4^ however, its enzymatic activity remains controversial to date. Among the reported substrates of ALKBH1 are different types of methylated nucleotides of DNA^5,6^ or RNA,^7,8^ methylated lysine of histone H2A,^9^ as well as abasic sites of DNA.^10^ Meanwhile, the functional importance of ALKBH1 in embryogenesis has been demonstrated in *Alkbh1* knockout mice from several independent studies, suggesting a distinct role of ALKBH1 in gene regulation and early development.^11–13^

DNA *N*^6^-deoxymethyladenosine (*N*^6^-mA) was recently identified in eukaryotic genomes,^6,14–21^ expanding the repertoire of epigenetic marks. In mouse embryonic stem cells (mESCs), *N*^6^-mA was shown to enrich at young LINE-1 transposon and its deposition correlates with epigenetic silencing.^6^ In vertebrates, *N*^6^-mA was further shown to accumulate to high abundance (~0.1%–0.2% *N*^6^-mA/dA) after fertilization and diminish to the background level (< 0.001%) with the progression of the embryo development.^15^ Besides, higher level of *N*^6^-mA was also detected in mouse brain upon environmental stress^22,23^ as well as the highly malignant brain cancer glioblastoma,^17^ all of which suggest a process of active regulation of *N*^6^-mA. Several studies including ours demonstrated that ALKBH1 is the demethylase of *N*^6^-mA on ssDNA;^6,16–18,24^ however, the exact biochemical activity of ALKBH1 and the underlying molecular basis remains unclear so far.

Here, we report the biochemical, structural and genomic studies of DNA *N*^6^-mA demethylation by ALKBH1. By establishing a reliable in vitro enzymatic assay, we demonstrated that ALKBH1 prefers bubbled and bulged DNAs, instead of ssDNA or dsDNA substrates. We further generalized the favorable substrates of ALKBH1 as nucleic acids that share a locally unpairing feature with flanking duplex(es), such as D-loop, R-loop, and DNA or RNA stem loop. Structural studies of ALKBH1 in substrate-free and a 21-mer bulge DNA-bound state revealed the molecular determinants underlying the observed substrate preference, including unique features of a stretch-out Flip1 motif and a functionally indispensable N-terminal “α1” helix. Furthermore, ssDNA-seq and DIP-seq analyses revealed significant co-localization of *N*^6^-mA with base unpairing regions in mouse early developmental cell line, highlighting a role of mammalian ALKBH1 in dynamic genomic regulation.

## Results

### ALKBH1 prefers bubbled DNAs as substrate instead of ss-/ds-DNAs

**Fig. 1:**
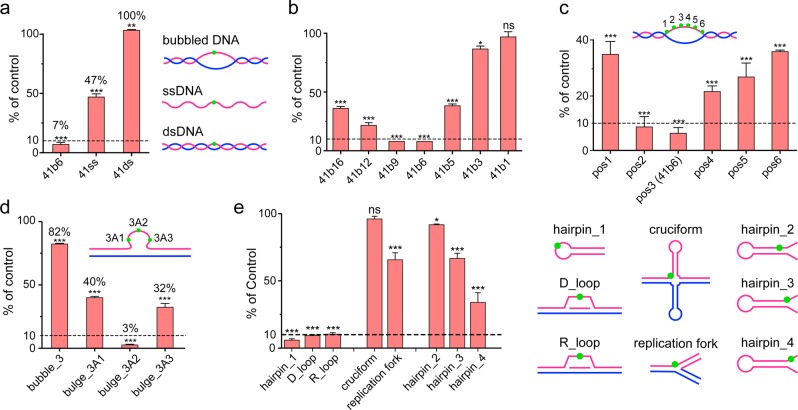
ALKBH1 prefers locally unpaired DNAs as substrates, but not ss-/ds-DNAs. **a**–**e** LC-MS/MS analyses of nucleoside hydrolytes for in vitro *N*^6^-mA demethylation assays using purified mouse ALKBH1 in full length and various DNA substrates. **a** 41-nt ssDNA (41ss), 41-bp dsDNA (41ds), and 41-bp dsDNA with a 6-nt mismatched region in the middle to mimic a bubble (41b6); **b** 41-bp bubbled DNAs, varied in the bubble size from 1 to 16 nt; **c** 41-bp bubbled DNAs with *N*^6^-mA in different position of a 6-nt bubble (pos1-6); **d** *N*^6^-mA in the middle of a 3-nt bubble (bubble_3) and different position of a 3-nt bulge (bulge_3A1, 3A2, and 3A3); **e** DNAs with different secondary structures. Substrates were shown as cartoon and *N*^6^-mA was shown as a green dot. Relative amounts of *N*^6^-mA were calculated for each measurement according to standard curves of *N*^6^-mA nucleoside. Demethylation results were reported as residual *N*^6^-mA compared to its blank control (sample without ALKBH1). *, **, ***, and ns indicate *P* *<* 0.05, 0.01, and 0.001 and ≥ 0.05, respectively, *t*-test; error bars, ± SD of three biological replicates

To explore the demethylation activity of ALKBH1, we first established a stable in vitro enzymatic assay based on LC-MS/MS (Supplementary information, Fig. S1a–c). Sequences used in our study were synthesized according to the known *N*^6^-mA-containing sequences found in mESCs^6^ (Supplementary information, Table S1). Consistent with the previous study,^6^ full-length ALKBH1 was able to catalyze the demethylation of *N*^6^-mA of ssDNA but not dsDNA (Fig. 1a). However, the demethylation fraction of ssDNA can only reach about 50% despite extensive efforts in reaction condition optimization (data not shown), suggesting that ssDNA may not be the optimal substrate. The preference of ALKBH1 to ssDNA prompted us to inspect its activity towards bubbled DNA, a form of non-B DNA structure with regulatory potential in genome.^25^ Remarkably, ALKBH1 displayed pronounced *N*^6^-mA demethylation activity over a 6-nt (nucleotide) DNA bubble compared to ssDNA and dsDNA (Fig. 1a). Optimal activities (demethylation fraction > 90%) were observed for 6- and 9-nt bubbles on the 41-bp dsDNA templates, and this activity dropped dramatically when the bubble size was shrunk to 3 nt or less (more dsDNA-like) and reduced gradually following bubble extensions to 16 nt (more ssDNA-like) (Fig. 1b). To explore the sequence dependence, three more oligos mimicking the natural sequences identified by SMRT-ChIP-seq analyses^6,18^ were screened for demethylation activity; *N*^6^-mA in all tested 6-nt bubbled DNA were efficiently demethylated regardless of the sequence motif (Supplementary information, Fig. S2a).

We also demonstrated that the removal of *N*^6^-mA from bubbled DNA is strictly dependent on Fe(II) and 2-oxoglutarate (Supplementary information, Fig. S2b). Alanine mutation of two conserved ion-coordinating residues, H231 and D233, completely abolished the activity (Supplementary information, Figs. S1d, e, S2c).

### Substrates of ALKBH1 share a locally unpairing feature

We further interrogated the substrate preference of ALKBH1 by changing the position of *N*^6^-mA in the 6-nt bubble (Fig. 1c). We observed that *N*^6^-mA was more efficiently removed when placed at the 2nd and 3rd positions from the 5′-end. We hypothesized that *N*^6^-mA at these positions is more flexible to flip out for demethylation. In this respect, we designed a 3-nt bulged DNA, which forces nucleotides in the bulge to flip out. Notably, the middle *N*^6^-mA in the 3-nt bulge was efficiently demethylated compared to that in the 3-nt bubble (97% vs. 18% demethylation fraction, Fig. 1d), and the 1st and 3rd *N*^6^-mAs, which are less flexible than the middle one, were shown to be more resistant to demethylation. These results stressed that the flipping-out of *N*^6^-mA is crucial to promote its demethylation by ALKBH1.

In parallel, we also examined *N*^6^-mA demethylation of ALKBH1 towards other reported secondary structures of our genome.^25,26^ *N*^6^-mAs deposited in the flexible region of hairpin (DNA stem loop), D-loop and R-loop were efficiently demethylated as in bubble and bulge, while those in cruciform and replication fork, which are locally more restricted, were less demethylated (Fig. 1e). In support, we generated three additional hairpins by placing *N*^6^-mA at the paired stem, branch point, and the unpaired tail, respectively, and found that *N*^6^-mA became more prone to be demethylated as its flexibility increased (Fig. 1e).

RNA oligos with *N*^6^-methyladenosine (m6rA) were also synthesized for demethylation test. We showed that m6rA in RNA hairpin, RNA-RNA bubble and RNA-DNA hybrid bubble can also be demethylated by ALKBH1 (Supplementary information, Fig. S2d). Another two RNA modifications, 5-methylcytosine (m5rC) at anticodon loop^7^ and 1-methyladenosine (m1rA) at TΨC loop^8^ from tRNA were also reported as substrates of ALKBH1, both of which locate at regions corresponding to the loop region of substrates mentioned above. In our assay, both m5rC and m1rA synthesized as reported can be demethylated by ALKBH1 to various degrees; however, m1rA in bubbled RNA (RNA-RNA and RNA-DNA hybrid) as well as 1-methyldeoxyadenosine (1 mA) in ss-, ds-, and bubbled DNA were more resistant to demethylation (demethylation fraction < 20%) (Supplementary information, Fig. S2d). It is noteworthy that all potential substrates of ALKBH1, as we have shown above, share a locally unpairing feature in which the modified nucleotide is prone to flipping out for demethylation.

The subcellular localization of ALKBH1 is also under debate. Although in certain cell lines, such as HeLa cells^5^ and HEK293 cells,^7,27^ ALKBH1 was suggested to localize and function in mitochondria. Studies in mouse trophoblasts,^11^ human and mouse ESCs,^6,9^ human mesenchymal stem cells^28^ and human cancers,^17^ all demonstrated its localization in nucleus and functions. Consistent with our previous works,^6,17^ we showed that ALKBH1 is located in nucleus in mESC, and its localization was not influenced with fusion tag switched from N- to C-terminal (Supplementary information, Fig. S2e). Thus, we focus on ALKBH1 function as genomic DNA *N*^6^-mA demethylase in the current study.

### Detection of *N*^6^-hydroxymethyladenine (*N*^6^-hmA) during *N*^6^-mA demethylation by ALKBH1

**Fig. 2:**
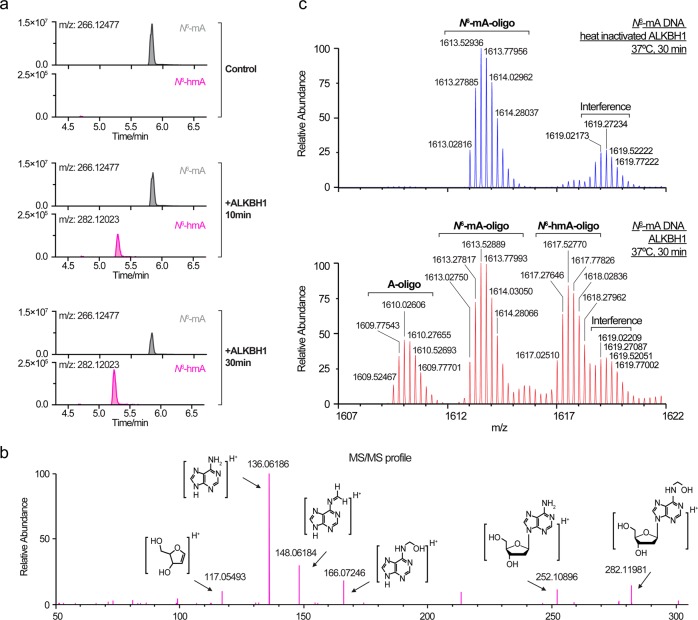
Formation of *N*^6^-hmA during oxidative demethylation of *N*^6^-mA in DNA by ALKBH1. **a, b** Demethylation products analyzed as digested nucleosides. **a** Chromatograms of deoxyadenosine derivatives. An 18-bp bulged DNA with one *N*^6^-mA modification (1.5 μM) was treated by 1.5 μM of ALKBH1 for 10 and 30 min, enzymatic digested to nucleosides, and analyzed by UHPLC-Orbitrap MS. The control group was treated with 1.5 μM of heat-inactivated ALKBH1 (incubated at 80 °C for 10 min before added into reaction) for 30 min and analyzed in the same way. The peaks of *N*^6^-mA and *N*^6^-hmA nucleoside were extracted with m/z = 266.12477 and m/z = 282.11968. **b** MS/MS spectrum of putative *N*^6^-hmA (**a**, +ALKBH1, 30 min) acquired from HCD fragmentation of Orbitrap mass spectrometer. The assigned fragment structures were generated using ChemDraw Professional 16.0 (CambridgeSoft). **c** Mass spectra of demethylation products analyzed as undigested oligos. Bulged DNA in reaction and control group was treated as described above but analyzed as undigested oligos by UHPLC-Orbitrap MS (see methods). All ions were detected as adducts of [M-4H]^4-^. The theoretical m/z values of *N*^6^-mA-oligo, dA-oligo and *N*^6^-hmA-oligo were 1613.02518, 1609.52127 and 1617.02391, with mass error 1.4 p.p.m., 2.1 p.p.m., and 0.7 p.p.m., respectively. Monoisotopic peaks corresponding to *N*^6^-hmA-oligo and dA-oligo were observed after treated by active ALKBH1 for 30 min

To investigate the demethylation process of ALKBH1, we monitored the reaction with online LC-MS/MS to detect *N*^6^-hmA, an expected intermediate of *N*^6^-mA oxidized by Fe(II)/α-KG-dependent dioxygenase. First, we analyzed the demethylation products in the form of digested nucleosides as we did above (Methods). A peak corresponding to *N*^6^-hmA (m/z = 282.12023, with mass error 0.3 ppm) was detected 10 min after reaction started, which was not found when the *N*^6^-mA substrate was incubated with the heat-inactivated ALKBH1 (Control). The quantity of *N*^6^-hmA substantially accumulated as reaction proceeded (30 min), while the peak corresponding to *N*^6^-mA (m/z = 266.12477) gradually decreased (Fig. 2a). In support, the MS/MS spectrum of potential *N*^6^-hmA peak (30 min) was acquired, and the putative fragments were assigned, confirming the presence of *N*^6^-hmA (Fig. 2b). Unlike the FTO-mediated m6rA oxidation,^29^ no confident peak corresponding to *N*^6^-formyladenosine (*N*^6^-fA) was observed in our study of ALKBH1. The intermediate was further confirmed based on the intact oligo (undigested) analysis using UHPLC-Orbitrap MS (Methods). Consistently, the peak corresponding to *N*^6^-hmA-oligo, the expected oxidation product of *N*^6^-mA-oligo, was observed at the endpoint of the reaction, while no comparable *N*^6^-fA-oligo signal was identified (Fig. 2c). Remarkably, the *N*^6^-hmA-oligo signal dropped to background level after overnight incubation at 10 °C following the stop of enzymatic reaction at 30 min, suggesting spontaneous decomposition of *N*^6^-hmA to A independent of ALKBH1 catalysis under the reaction condition (pH 8.0) (Supplementary information, Fig. S3). During the preparation of our manuscript, the existence of *N*^6^-hmA in mammalian genomes as well as its accumulation in lung carcinoma tissues was reported and ALKBH1 was shown to convert *N*^6^-mA to *N*^6^-hmA in vivo.^30^ With these intriguing results, further studies are needed to delineate the biological function and regulatory roles of *N*^6^-hmA generated by ALKBH1.

### Overall structure of ALKBH1

**Fig. 3:**
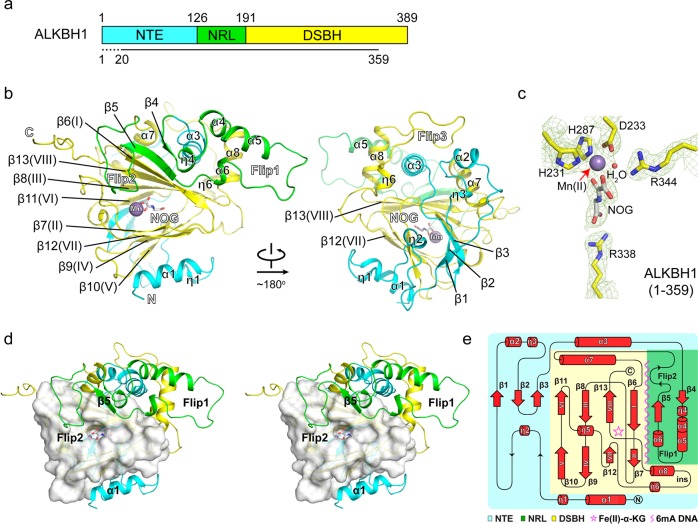
Overall structure of mouse ALKBH1 bound to Mn(II) and NOG. **a** Schematic domain architecture of ALKBH1. The N-terminal extension (NTE, residue 1–125), nucleic acid recognition lid (NRL, residue 126–190), and double-stranded β helix (DSBH, residue 191–389) are colored in cyan, green, and yellow, respectively. ALKBH1_1–359_ was used for crystallization and residues 1–19 were not modeled due to flexibility, indicated as dashed line. **b** Ribbon view of ALKBH1 in two orientations. The color coding of domains is the same as in **a**. NOG is shown in stick representation. Mn (II) is shown as a gray ball. **c** Close-up view of the catalytic center of ALKBH1. Light green meshes: 2Fo-Fc omit maps contoured at 1.5 σ level. **d** Stereoview diagram showing spatial connection of DSBH with α1 helix and NRL (Flip1 and Flip2 connected by β5). DSBH is shown as white surface, and other elements are shown as color-coded ribbon. **e** Topology of ALKBH1. Cofactors are denoted by a magenta star. The substrate-binding surface is indicated by a magenta wavy line

Mouse ALKBH1 protein has 389 residues and consists of an N-terminal extension (NTE), a nucleotide-recognition lid (NRL), and a C-terminal double-stranded β helix (DSBH) domain (Fig. 3a). To elucidate the molecular mechanisms underlying ALKBH1 function, we determined the 2.5 Å crystal structure of mouse ALKBH1 (residues 1–359, ALKBH1_1–359_) bound to Mn(II) and N-oxalylglycine (NOG), the substitutes for the reactive Fe(II) and α-KG (Fig. 3b, c, data collection and refinement statistics summarized in Supplementary information, Table S2). Residues 20–358 were modeled based on the electron densities. The DSBH catalytic core of ALKBH1 adopts a typical eight-stranded (I–VIII) jelly-roll fold that contains a major sheet (βI, βVIII, βIII, βVI) and a minor sheet (βVII, βIV, βV and βII) to sandwich Mn(II) and NOG at the active center. The central jelly-roll fold is notably wrapped around and stabilized by additional elements from NTE, NRL and DSBH (Fig. 3d, e). In particular, an N-terminal α1 helix of NTE buttresses the minor sheet from the bottom; an extended “coil-sheet” structure of NTE extends the β(V-VI) edge from the back; α3 of NTE and α7 of DSBH organize a helical cluster involving elements α2 and α8, and stack against the major sheet from the top. Moreover, the NRL subdomain composed of elements Flip1 and Flip2 attaches to the β(I) edge of the major sheet, and functionally constitutes the substrate recognition surface along with α1 helix (Supplementary information, Fig. S4).

### An extra “α1 helix” stabilizes the active center of ALKBH1

**Fig. 4:**
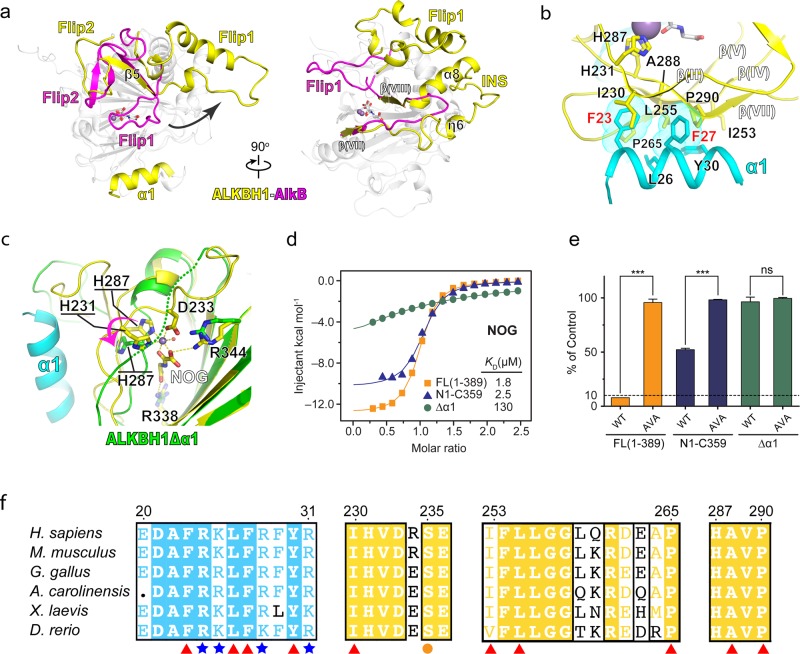
Role of α1 in active site integrity maintenance of ALKBH1. **a** Structural comparison of ALKBH1 and AlkB. α1, Flip1, Flip2, and a long insertion between β(VII) and β(VIII) (INS) of ALKBH1 are colored yellow; Flip1, Flip2, and a loop segment between β(VII) and β(VIII) of AlkB are colored magenta; the rest of ALKBH1 and AlkB are colored gray. **b** Hydrophobic interactions of α1 with the minor sheets of ALKBH1. α1 and the minor sheets are colored cyan and yellow, respectively. Key hydrophobic residues are shown as stick. Purple ball, Mn (II); white stick, NOG. **c** Structural comparison of ALKBH1_1–359_ and ALKBH1_37–369_ (ALKBH1Δ α1) around the catalytic center. α1 is colored cyan. The DSBH core of ALKBH1_1–359_ and ALKBH1_37–369_ is colored yellow and green, respectively. Key residues involved in cofactor binding are shown as sticks. Dotted line, the disordered β(II) that comprises H231 and D233 of ALKBH1Δ α1. **d** Overlay of ITC fitting curves of ALKBH1 in full length (orange), ALKBH1_1–359_ (purple) and ALKBH1Δ α1 (green) titrated with NOG, respectively. **e** In vitro *N*^6^-mA demethylation assay using ALKBH1 in full length, ALKBH1_1–359_ and ALKBH1Δ α1 towards 41b6. Both wild-type and H231A/D233A double mutated protein of all three constructs were included, respectively. Color codings are the same with **d**. ***, and ns indicate *P* *<* 0.001 and ≥ 0.05, respectively, *t*-test; error bars, ± SD of three biological replicates. **f** Protein sequence alignment of ALKBH1 among vertebrates. Red triangles, residues participate in hydrophobic interaction; blue stars, positive charged residues on α1; yellow circle, a key residue involved in modified base interaction

Although ALKBH1 is much longer than its bacterial homolog AlkB (389 vs. 216 residues, Supplementary information, Fig. S5), the catalytic core of ALKBH1 superimposed well with bacterial AlkB,^31,32^ with a root mean square deviation (r.m.s.d) of 0.759 Å over 105 Cα atoms. In contrast, three notably unique structural features to ALKBH1 arise outside the core structure: a far N-terminal α1 helix packed aside the catalytic center, the stretch-out of an extended Flip1, and a long insertion between β(VII) and β(VIII) (INS) that stabilizes Flip1 through helical cluster formation (Fig. 4a). Such features hold unique for ALKBH1 when comparing with all structures of its human paralogues determined so far, including ALKBH2, 3, 5, 7, 8 and FTO^32–39^ (Supplementary information, Fig. S6).

The extra α1 helix forms extensive hydrophobic contacts with the minor sheet involving residues F23, L26, F27, Y30 of α1, I230 of β(II), P290 of β(VII), and I253, L255 of β(IV), and sequence alignment shows that these residues are highly conserved in vertebrates (Fig. 4b, f). The α1 helix is critical to maintain the structural integrity of the active center; in particular, F23 and F27 directly stabilize the minor β sheet through extensive hydrophobic contacts. In support, the structure of an N-truncated ALKBH1 (residue 37–369, ALKBH1Δα1) determined at 2.3 Å by the Se-SAD method (Supplementary information, Table S2) demonstrates that missing of α1 helix (residues 22–32) results in distortion of the co-factor coordination geometry as reflected by flipping away of H287 and disordering of β(II) that comprises H231 and D233 (Fig. 4c). Neither Mn(II) nor NOG was observed in the crystal (Supplementary information, Fig. S7a), and the impaired co-factor coordination of ALKBH1Δα1 in solution was also confirmed by calorimetric titration (Fig. 4d). Furthermore, enzymatic assays showed that ALKBH1Δα1 was totally inactive in *N*^6^-mA demethylation (Fig. 4e). Although an α1-truncated human ALKBH1 protein was reported to have demethylation activities towards m1rA in tRNA,^8^ we performed in vitro enzymatic assay using the α1-truncated human ALKBH1 and hardly detected any activities towards m1rA in tRNA stem loop nor *N*^6^-mA in DNA bubble (Supplementary information, Fig. S7b, c), further underlying the importance of α1 helix.

### A “stretch-out” Flip1 and “α1” form a unique substrate channel of ALKBH1

**Fig. 5:**
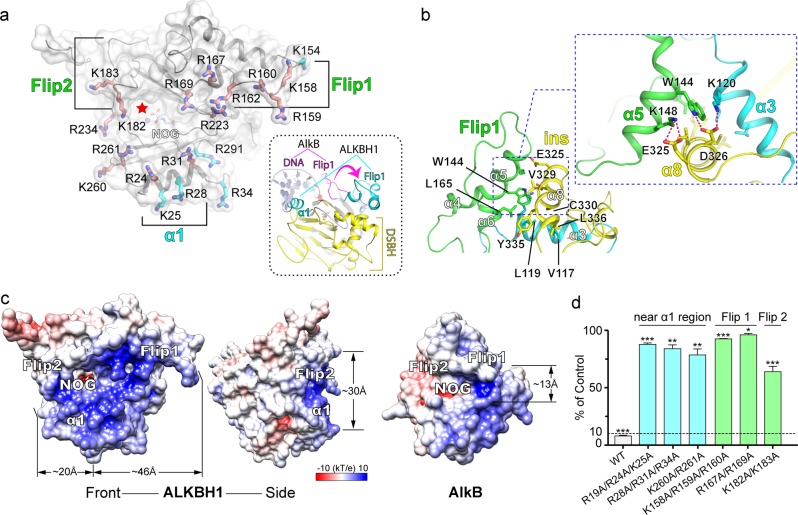
Substrate-binding surface analysis and mutagenesis studies. **a** Distribution of basic residues along α1, Flip1 and Flip2 regions of ALKBH1_1–359_. Lysine and arginine are shown in stick. Residues critical to enzyme activity, revealed by mutagenesis experiments, are colored in deep salmon; residues shown to be less indispensable are colored cyan. The catalytic center is indicated by a red star. Right panel, structural comparison of ALKBH1_1–359_ and AlkB-DNA (PDB: 3BI3) complex. α1 and Flip1 of ALKBH1 are colored cyan; DSBH of ALKBH1 is colored yellow. Flip 1 of AlkB is colored magenta; DNA of AlkB and other parts of both proteins are colored gray. **b** Hydrophobic and hydrogen-bonding interactions among Flip1, α3 (from NTE), and ins. Flip1, α3 and ins are colored green, cyan, and yellow, respectively. Residues involved in hydrophobic and hydrogen-bonding interaction are shown as stick. **c** Comparison of electrostatic potential surface of ALKBH1 and AlkB. Both surfaces are colored as a spectrum of the surface electrostatic potential ranging from blue (10 kT/e) to red (−10 kT/e). **d** In vitro demethylation assays of ALKBH1 mutants towards the 41b6 DNA substrate

The NRL subdomain, further composed of two sections named Flip1 and Flip2,^40^ is a unique feature of the AlkB family dioxygenases.^31,37^ Unlike the highly conserved catalytic core, the NRL subdomain is structurally variable among AlkB members and has been shown to play a critical role in substrate interaction.^31–35,37–39,41^ In most ALKBH1 homologs,^31–33,37,41,42^ the Flip1 motif adopts a bending conformation covering the active center (Supplementary information, Fig. S6), and forms a positively charged groove for nucleic acid binding (Fig. 5c, right). In the case of AlkB and ALKBH2, Flip1 has been shown to facilitate the recognition of modified base as well as base flipping for demethylation.^32,42^ In contrast, Flip1 of ALKBH1 assumes a fully extended conformation (Fig. 5a), which is stabilized by extensive hydrophobic and hydrogen-bonding interactions through helical cluster formation among α3 and an “INS” motif, a non-conserved long insertion between β(VII) and β (VIII) unique to ALKBH1 (Fig. 5b; Supplementary information, Fig. S6). Conceivably, the stretch-out of Flip1 likely leads to the lack of autonomous base-flipping activity of ALKBH1, thus accounting for the observed incompetence of dsDNA demethylation by ALKBH1. By contrast, the locally unpaired DNA bubble can well serve as substrate for ALKBH1 since base flipping has been primed for direct recognition and catalysis.

On the other hand, considerable lysine and arginine residues are enriched within Flip1, which constitute one extended and electrostatically positive wing around the catalytic cleft (Fig. 5a, c). In parallel, an additional string of basic residues was found around α1 helix and Flip2, forming the other positive wing of the substrate channel (Fig. 5a, c). The dimension of the substrate channel is ~66 Å in length and ~30 Å in diameter (Fig. 5c, left and middle), which is well-positioned for duplex substrate recognition of ~20 bp in length (contour length = 68 Å for B-form DNA). By contrast, AlkB has a relatively narrow binding groove of ~13 Å in diameter (Fig. 5c, right), consistent with its preference for ssDNA substrates.^32,42^

We next performed mutagenesis studies to explore the importance of the basic residues around the substrate channel. Alanine mutation of basic residues of Flip1 region (K158A/R159A/R160A, R167A/R169A) dramatically abolished *N*^6^-mA demethylation activity of ALKBH1 (Fig. 5d), and electrophoretic mobility shift assays further demonstrated impaired DNA binding of these mutants (Supplementary information, Fig. S7d). As well, the basic residues of the α1 and Flip2 regions were similarly verified to be indispensable for substrate binding and enzymatic activities (Fig. 5d; Supplementary information, Fig. S7d). Hence, the above data underscore a role of those basic residues for nucleic acid substrate recognition. In the meantime, the K/R-rich substrate-binding surface less likely supports the positively charged histones as substrates for ALKBH1.^9^

### Co-crystal structure of ALKBH1 in complex with bulged DNA

**Fig. 6:**
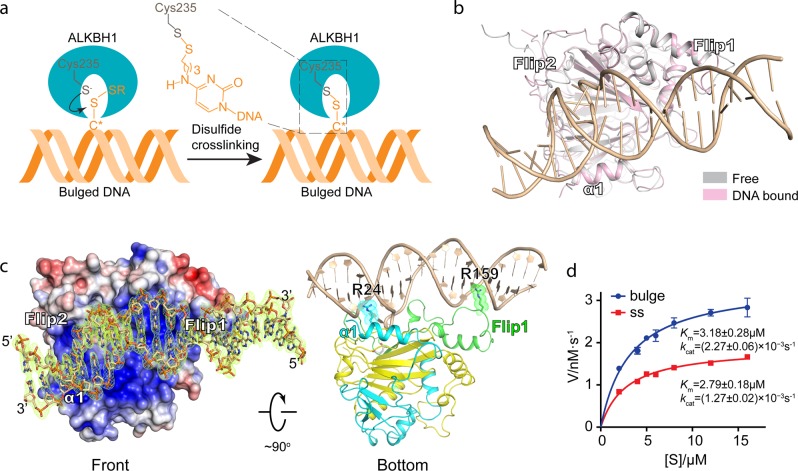
Structure of ALKBH1 in complex with a 21-mer bulged DNA. **a** Diagram of the cross-linking strategy. A disulfide bond can be formed between C* in the bulge and Cys235 in the active site of ALKBH1. **b** Structural alignment of ALKBH1_20–355_ in complex with bulged DNA and ALKBH1_1–359_. **c** Left, ALKBH1 in complex is shown as electrostatic potential surface in the front view, colored as a spectrum of its surface electrostatic potential ranging from blue (10 kT/e) to red (−10 kT/e). The bulged DNA is shown as sticks with the 2Fo-Fc omit map contoured at 2.0 σ. Right, cartoon of ALKBH1 in complex with bulged DNA in bottom view. NTE, NRL, and DSBH are colored cyan, green, and yellow, respectively. The bulged DNA is colored wheat. R24 and R159 are shown as sticks and space-filling dots. **d** Michaelis–Menten plots of the steady-state kinetics of mouse ALKBH1-catalyzed *N*^6^-mA demethylation in bulged and ssDNA. *N*^6^-mA-containing bulged/ss DNAs at various concentration were incubated with 1.5 μM of mouse ALKBH1. Demethylation products were analyzed as undigested oligos (see methods) by UHPLC-Orbitrap MS. Relative product amounts were calculated according to standard curve of dA-DNA oligo. The initial velocity (*V*_o_) was calculated based on the dA-DNA generated in the assumed linear interval of the first 2.5 min. Less than 20% of the substrate was consumed in all reactions. The kinetic parameters, *K*_m_ and *k*_cat_ were generated by GraphPad software with the Michaelis–Menten equation. Error bars, SD of biological triplicates

To further explain the preference of ALKBH1 for locally unpairing DNAs, we determined the 2.4 Å crystal structure of ALKBH1 bound to a 21-mer bulged DNA (data collection and refinement statistics summarized in Supplementary information, Table S2). To obtain a stable and homogenous complex sample for crystallization, we adopted a disulfide cross-linking strategy by introducing a cysteine residue (S235C) in the catalytic pocket and a disulfide-modified cytosine within the bulge (Fig. 6a and methods). The overall structure of DNA-bound and the free state ALKBH1 superimposed well (r.m.s.d_Cα_ = 0.555 Å), except for conformational adjustments of several flexible loops of Flip2 and Flip1 regions (Fig. 6b). As expected, the structurally stabilized Flip1 element (Fig. 5b) remains in a “stretch-out” conformation even in the presence of DNA substrate, lending further supports to the lack of autonomous base-flipping capability of ALKBH1.

Consistent with our previous analysis, the 21-mer bulged DNA substrate is docked onto a wide and positively charged concave surface in between Flip1, Flip2 and α1 (Fig. 6c, left). The bulged DNA adopts a B-form structure with both strands contributing to enzyme-substrate recognition primarily through “basic-backbone” interactions. Notably, two arginine residues (R24 of α1 and R159 of Flip1) insert into DNA minor grooves from both ends and function as two fingers to hold the DNA substrate (Fig. 6c, right). Such a recognition mode likely helps to orientate the DNA substrate for proper insertion of the flipped base into the active pocket, which is supported by dramatic activity loss of the R24A and R159A mutants (Supplementary information, Fig. S7e). The observed interaction mode between ALKBH1 and bulged DNA underscores the need of both the flanking duplex/stem region and the flipped base for recognition and catalysis. In support, enzymatic kinetics analyses showed that ALKBH1 displayed higher catalytic efficiency towards bulged DNA (*k*_cat_*/K*_m_ = 0.71 × 10^3^ M^−1^ s^−1^) than ssDNA (*k*_cat_*/K*_m_ = 0.46 × 10^3^ M^−1^ s^−1^) (Fig. 6d). Interestingly, the calculated apparent *K*_m_ values are comparable for both ssDNA and bulged DNA (2.79 vs. 3.18 μM). By contrast, the *k*_cat_ value is higher for bulged DNA (1.27 × 10^–3^ vs. 2.27 × 10^–3^ s^−1^), which suggests that the duplex regions of the bulged DNA substrate promote efficient enzymatic turnover likely by facilitating optimal alignment of the flipped *N*^6^-mA in the catalytic center.

Unlike ALKBH1, both AlkB and ALKBH2 showed comparable activities on ss-, ds-, and bubbled substrates owing to the existence of a bending Flip1 for base flipping and a relatively narrow basic groove for nucleic acid strand recognition (Supplementary information, Fig. S8). Collectively, our complex structural study demonstrated a distinct substrate recognition mode of ALKBH1 as compared to other AlkB family members, which ultimately determines the unique preference of ALKBH1 for base unpairing DNA.

### Substrate-binding pocket of ALKBH1

**Fig. 7:**
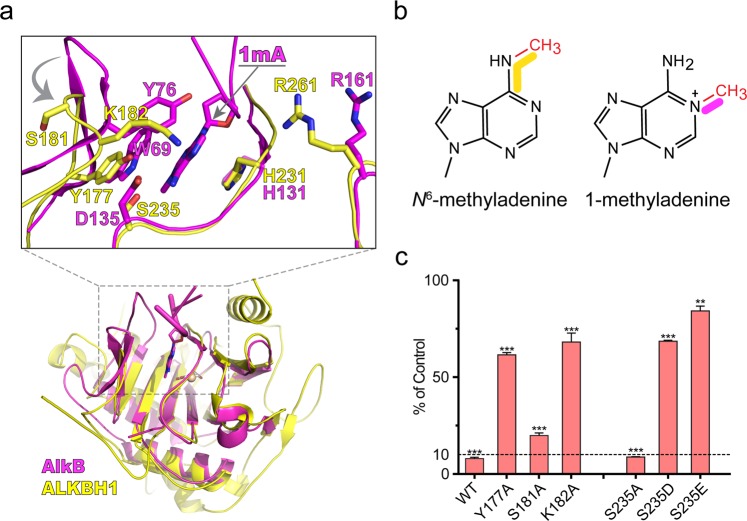
Base recognition pocket of ALKBH1. **a** Structural comparison reveals high similarity between ALKBH1 (yellow) and AlkB (magenta). Critical *N*^6^-mA recognition residues of the catalytic pocket (shown in stick) are revealed by structural alignment of ALKBH1 and AlkB. **b** Chemical structures of *N*^6^-methyladenine and 1-methyladenine. Yellow and magenta denote the branching of the methyl group of *N*^6^-methyladenine and 1-methyladenine, respectively. **c** In vitro demethylation assays of ALKBH1 mutants of residues shown in **a** towards 41b6. ** and *** indicate *P* *<* 0.01 and 0.001, respectively, *t*-test; error bars, ± SD of three biological replicates

Substrate-binding pocket of AlkB family members is formed by residues from NRL subdomain and DSBH fold, in which a target base is inserted, with its alkyl group pointing to the active site.^31,32,41,42^ Substrate-binding pocket of ALKBH1, compared to those of its homologs, was shown to be more similar to that of AlkB: comprising residues from DSBH fold (βI-III, βVIII and a loop between βII-III) assume nearly identical conformation in both proteins. Major differences come from the NRL subdomain, with a shorter Flip2 from ALKBH1 showing a “back-drawing” conformation, and its “stretch-out” Flip1 resulting in a more exposed pocket (Figs. 4a and 7a).

At this stage, covalent crosslinking of the flipped base with ALKBH1 within the active pocket prevents us from in-depth analysis of methyl base recognition in a native state. We then turn to structure-based comparison and mutagenesis studies to explore the methyl base-binding pocket of ALKBH1. Structural alignment of ALKBH1_1–359_ with AlkB bound to its 1 mA substrate revealed potential residues involved in *N*^6^-mA interaction of ALKBH1 (Fig. 7a). In particular, Y177 and H231 of ALKBH1 overlap with W69 and H131 of AlkB, and likely play a similar role in sandwiching *N*^6^-mA for demethylation; S235 of ALKBH1 overlaps with D135 of AlkB, and is located next to the hydrogen-bonding edge of the modified base. In AlkB, D135 has been shown to modulate base selectivity,^31,42–44^ and D135S or D135A mutations disrupted enzyme activity to its natural substrate 1 mA due to loss of favorable interactions.^42,44^ Sequence alignment of ALKBH1 orthologues revealed that S235 is conserved among vertebrates (Fig. 4f). Considering that the dimension of *N*^6^-mA is bulkier than 1 mA (Fig. 7b), a shorter side chain of S235 may endow additional space for substrate accommodation. In support, S235A mutation did not affect ALKBH1 activity; while S235D and S235E resulted in a dramatic activity loss likely due to introduced steric clashes, especially in the case of S235E (Fig. 7c). We also tested the enzymatic activities of Y177A, S181A, and K182A, and revealed that Y177A, K182A but not S181A displayed clear activity decrease, consistent with a role of Y177 and K182 but not S181 in direct substrate pocket formation (Fig. 7c).

### *N*^6^-mA co-localizes with genomic unpairing regions during TSC development

**Fig. 8:**
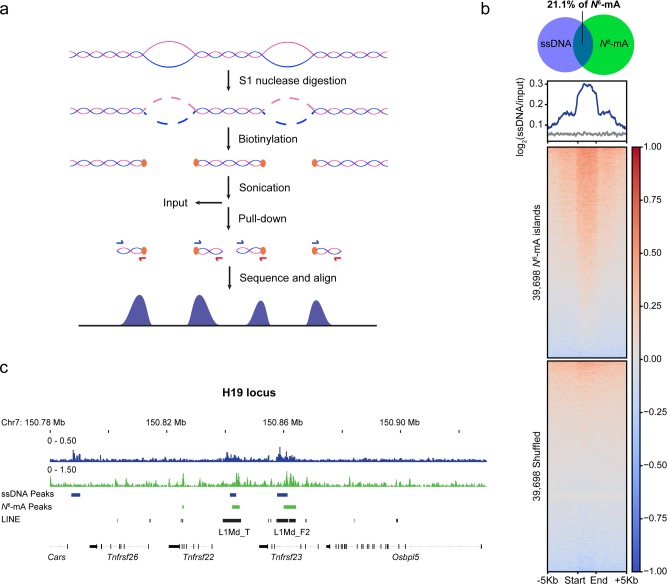
Colocalization of *N*^6^-mA and ss-DNA region in mouse genome. **a** General flow of ssDNA-seq. **b** Enrichment of ssDNA in *N*^6^-mA genomic regions. Top: overlap between regions reported as a percentage of *N*^6^-mA peaks in LV1 cells (mESCs undergoing cell fate transition) that are intersected by ssDNA peaks (*P* < 2.2 × 10^–16^, Fisher’s exact test). Bottom: aggregate profiles and heatmaps for ssDNA-seq signal over *N*^6^-mA regions. **c** ssDNA-seq tracks at a representative genomic region (H19 locus) that are enriched for *N*^6^-mA

A recently developed single-stranded DNA sequencing approach based on permanganate and S1 nuclease mapping (ssDNA-seq) successfully detected ssDNA regions (not single-stranded DNA) as a common feature across genome of activated B cells.^25^ We validated the ssDNA-seq approach in mouse ESCs using a wild-type line (TT2) which has low *N*^6^-mA levels in the self-renewing state (Fig. 8a and Methods). Totally, 8134 ssDNA peaks (false discovery rate cutoff = 0.01) were identified in mouse ESCs (Supplementary information, Fig. S9a). Owing to the more stringent peak calling with an input control utilized in this study, this number is expectedly less than that identified in activated B cells. Still, the genomic distribution of the peaks was consistent with the previously published findings, with a majority of peaks within genes (56.3%, *P* < 4.9 × 10^–324^) and enrichment within 1 kb of transcription start sites (TSS; 1.7%, *P* = 2.4 × 10^–41^) (Supplementary information, Fig. S9a, b). As expected, ssDNA was present at actively transcribed pluripotency genes (e.g., *Nanog* and *Slc2a3*, Supplementary information, Fig. S9g).

We further interrogated whether *N*^6^-mA was present on the unpairing regions in mouse genome. As the genome-wide *N*^6^-mA level in mouse ESCs is too low (6–7 p.p.m)^6^, we turned to another early development system with upregulated *N*^6^-mA for co-localization analyses of *N*^6^-mA and ssDNA regions. Our most recent study observed a substantial upregulation of *N*^6^-mA level during the transition of mouse ESCs to trophoblast stem cells (TSCs) (in press). By applying ssDNA-seq to ESCs undergoing cell fate transition (named as “LV1” hereafter), we identified more than 67,000 ssDNA-enriched regions, a majority of which were within 1 kb of predicted SIDD regions (65.4%). Notably, ssDNA peaks in LV1 were detected primarily within distal intergenic regions (55.9%) while significantly depleted within genes (35.1%, *P* = 2.9 × 10^–320^) and near TSS (0.7%, *P* *=* 6.2 × 10^–74^) (Supplementary information, Fig. S9d, e), which is in contrast to the predominant gene-associated ssDNA regions in self-renewing ESCs (Supplementary information, Fig. S9a, b) and activated B cells.^25^ The genome-wide distribution of *N*^6^-mA in LV1 was interrogated in parallel using DNA immunoprecipitation by anti-*N*^6^-mA antibody followed by next generation sequencing (DIP-seq). Reads of DIP-seq (95.5%) were confidently mapped to the mouse genome rather than mitochondrial or genomic DNA from other species. Analyses on genome-wide level revealed that *N*^6^-mA peaks significantly intersect with ssDNA regions (21.1%, *P* *<* 2.2 × 10^–16^; Fig. 8b). Aggregation analysis further showed ssDNA enrichment within *N*^6^-mA peaks but not in adjacent or randomly shuffled regions (Fig. 8b). ssDNA region (referred to as scaffold/matrix attachment region-S/MAR previously^45^) was known to play an important role in gene expression and chromatin structure regulation at the H19 imprinting locus.^46^ At the H19 locus, in particular, our identified ssDNA peaks were shown to overlap with the well-known S/MAR regions identified by previous studies,^25,45^ with *N*^6^-mA also enriched in the region (Fig. 8c). Our results suggest the presence of *N*^6^-mA at locally unpairing regions in physiological conditions, which is consistent with the substrate preference of its demethylase.

## Discussion

Early studies on AlkB family members focused on its function in damage repair due to lack of knowledge in the occurrence and regulation of physiological DNA/RNA methylations and their derivatives.^5,47–50^ Despite being the founding member of its family, the cellular function of ALKBH1 has long been a debate in the field given its broad substrate spectrum beyond damage repair reported in different studies.^5–8^ Recently, ALKBH1 was reported as a *N*^6^-mA eraser of ssDNA substrate.^6^ Here, our biochemical profiling and structural analyses provide key evidence on novel features of ALKBH1 substrates, characterized by a locally unpairing structure that contains flipped *N*^6^-mA base with flanking duplex stems. The lack of autonomous base-flipping activity distinguishes ALKBH1 from other homologs such as ALKBH2^32^ and TET2^51^ that can directly demethylate dsDNA substrates. Our co-crystal structural studies demonstrated that the stretch-out conformation of a K/R-rich Flip1 creates a wide and basic concave surface best for duplex DNA engagement with the flipped methyl base inserted into the active center. The dimension of the substrate-binding surface of ALKBH1 well matches a B-form DNA of ~20 bp in length, which might serve as a physiological recognition unit centered on *N*^6^-mA-mediated epigenetic regulation.

The catalytic pocket of ALKBH1 is lined with a small side chain residue of S235 that is orthologously conserved in vertebrates, which allows an accommodation of the bulkier *N*^6^-mA base for catalysis, and thus plays a key role in modification base selectivity. In support, mutation of S235 to bulkier residues (Asp/Glu) disrupted *N*^6^-mA demethylation activity. Complete demethylation of *N*^6^-mA by Fe(II)/2-OG-dependent dioxygenases contains several catalytic steps.^50^ We were able to detect the existence of *N*^6^-hmA as an oxidized intermediate during *N*^6^-mA demethylation by ALKBH1. Our detection of *N*^6^-hmA, together with the published findings,^30^ suggesting that *N*^6^-hmA is a potential epigenetic mark reminiscent of 5hmC, and ALKBH1 may function as a key modulator of *N*^6^-mA and *N*^6^-hmA levels.

It has not escaped our attention that, in addition to *N*^6^-mA of unpairing DNA, ALKBH1 displayed in vitro activities towards m1rA, m6rA, m5rC to various degrees as long as a locally unpairing feature is retained. Similar broad substrate specificity has been observed for other AlkB family members. For example, structural and biochemical studies have established that bacterial AlkB is able to repair chemically diverse substrates different in base, alkylation position, and alkyl group.^31,43,44^ Comparing to its relatively broad base selectivity, ALKBH1 displays a high dependence on the secondary structure of nucleic acid substrates. Therefore, the biological function of ALKBH1 is likely regulated by its cellular localization and genomic targeting in cells. The reports of such additional activities to date are limited to certain cell lines, which have not been observed in developments or human diseases. Future studies will determine the physiological significance of those observations. Nonetheless, we observed dominant nuclear localization of ALKBH1 in mESCs, which, in concert with the reported cellular *N*^6^-mA demethylation activity,^6,16,17^ supports an important function of ALKBH1 in DNA *N*^6^-mA biology.

In living cells, the prevailing DNA conformation is the right-handed, double-stranded B-DNA. However, studies in the past decades have shown that our genome DNA is a dynamic molecule and is able to adopt various non-B structures, including melted bubbles in SIDD regions and DNA hairpins that share a locally unpairing feature.^25,45,52–54^ Formation of non-B DNA is influenced by either base sequence per se or biological processes that introduce local supercoiling. In turn, non-B DNAs usually play active roles in chromosome structure organization or gene regulation. For example, DNA bubbling is often coupled to topological stress occurring at locally AT-rich regions including S/MARs that are enriched with SIDD.^45,54–56^ In addition, nucleosome formation usually stabilizes negative supercoil with a superhelical density (σ) of about –0.07.^57^ Conceivably, in the processes like transcription or replication, nucleosome disassembly might facilitate ALKBH1 function by trigging local base unpairing. Here, we revealed co-localization of *N*^6^-mA and putative non-B DNA sites (ssDNA regions) in mouse genome. Our work provides an intriguing model for studying *N*^6^-mA demethylation on unpairing substrates in physiological environments, and further suggests that ALKBH1 is an important DNA demethylase associated with dynamic chromosome regulation.

## Materials and methods

### Protein expression and purification

The gene fragment encoding residues 1–389 (full length), 1–359 (ALKBH1_1–359_), 20–355 (ALKBH1_20–355_), and 37–369 (ALKBH1_37–369_) of mouse ALKBH1 were inserted into a modified pRSFDuet-1 vector, respectively. Point mutations were generated by a site-directed mutagenesis kit (Stratagene). Proteins with amino (N)-terminal His-tag were expressed in *Escherichia coli* strain BL21 (DE3) (Novagen) in the presence of 0.2 mM isopropyl-β-D-thiogalactopyranoside. Cells after overnight induction were collected by centrifugation and re-suspended in the lysis buffer: 20 mM Tris-HCl, pH 8.0, 100 mM NaCl. After cell lysis by an Emulsiflex C3 (Avestin) homogenizer, the centrifugation-cleared supernatant was applied to a HisTrap nickel column (GE Healthcare), and the bound protein was eluted with a linear imidazole gradient from 20 mM to 500 mM. The eluents were subjected to His-tag removal cleaved by PreScission protease, followed by ion-exchange chromatography on HiTrap QHP and HiTrap Heparin HP columns (GE Healthcare). Finally, the protein was purified to homogeneity through size exclusion chromatography on a Superdex 200 increase 10/300 column (GE Healthcare), and concentrated to 15 mg/mL in a buffer containing 20 mM Tris-HCl, pH 8.0, 100 mM NaCl for future use. Proteins used for enzymatic assays were purified in essentially the same procedures as described above. Of note, the full-length wild-type and mutated constructs of ALKBH1 do not contain PreScission protease cleavage site and the N-terminal His-tag was retained. Selenomethionine (SeMet)-labeled protein (ALKBH1_37–369_) was expressed in the methionine-auxotrophic strain B834(DE3) and purified using the same strategy as the native protein.

### Cross-linked ALKBH1-DNA sample preparation

To solve the crystal structure of ALKBH1-DNA complex, a slightly truncated frame of ALKBH1–ALKBH1_20–355_ was chosen for cross-linking and crystallization trials based on activity profiling and structural analysis. To generate the cross-linked ALKBH1_20–355_-DNA complex, the S235C mutation was introduced to the catalytic center for disulfide cross-linking, and other four C to S mutations (C300S, C304S, C129S, and C322S) were introduced to avoid non-specific cross-linking. The mutant protein was incubated with synthetic oligonucleotides (Supplementary information, Table S1) at 4 °C for 10 h, and further purified by Mono-Q anion exchange chromatography and size exclusion chromatography on a Superdex 200 increase 10/300 column (GE Healthcare).

### Crystallization, data collection and structure determination

Crystallization was performed via the sitting or hanging-drop vapor diffusion method under 4 °C by mixing equal volumes (0.2–1.0 µL) of the protein sample and the reservoir solution. Prior to crystallization, proteins were mixed with 2.3 mM MnCl_2_ and 4.3 mM NOG/succinic acid at a concentration of 10 mg/mL. SeMet-labeled ALKBH1_37–369_ crystals were grown in the solution containing 16% (w/v) PEG3350, 0.13 M ammonium citrate dibasic; native ALKBH1_1–359_ crystals were grown in the solution containing 10% (w/v) PEG6000, 0.1 M BICINE-HCl, pH 7.8; ALKBH1_20–355_-DNA complex crystals were grown in the solution containing 0.2 M NaCl, 0.1 M phosphate citrate, pH 4.2, 10% PEG3000. Crystals were briefly soaked in a cryo-protectant composed of reservoir solution supplemented with 20% glycerol, and then flash frozen in liquid nitrogen for data collection at 100 K. The data of SeMet-labeled ALKBH1_37–369_ was collected at wavelength of 0.9785 Å at Shanghai Synchrotron Radiation Facility beamline BL19U, and the data of native ALKBH1_1–359_ and ALKBH1_20–355_-DNA complex were collected at wavelength of 0.9792/0.9791 Å at Shanghai Synchrotron Radiation Facility beamline BL17U. Data were indexed, integrated and merged using the HKL2000 software package.^58^ Data collection statistics are shown in Supplementary information, Table S2.

The phase of ALKBH1_37–369_ was solved by the selenium single-wavelength anomalous dispersion method using PHENIX.^59^ The ALKBH1_1–359_ and the ALKBH1_20–355_-DNA complex structures were solved by molecular replacement using MOLREP.^60^ All structures were refined using PHENIX,^59^ with iterative manual model building using COOT.^61^ Model quality was analyzed with PROCHECK.^62^ Detailed structural refinement statistics are summarized in Supplementary information, Table S2.

### Preparation of nucleic acid substrates

All modified DNA/RNA oligos were purchased from Gene Link and the unmodified DNA/RNA oligos were purchased from GenScript. The ds-, ss-, bubbled DNA/RNA and stem-loop DNA/RNA were annealed at 25 μM in the annealing buffer (50 mM KCl, 4 mM MgCl_2_, 50 mM Tris-HCl, pH 8.0) using a thermocycler, during which the oligos were heated up to 95 °C for 2 min and gradually cooled to 25 °C over an hour. R-loop, D-loop, and replication folk were annealed according to the published method with minor modifications.^26^ Cruciform was generated by separately annealing the stem loop-containing forward and reversed strands as stated above, and then annealing both strands in equal molar ratio from 60 °C to 30 °C. All DNA and RNA substrates used in this study are summarized in Supplementary information, Table S1.

### In vitro demethylation assay

Demethylation assays were performed in 20 μL volume, which contains 30 pmol of annealed oligos and 4.08 μg of recombinant mouse ALKBH1 protein or mutant. The reaction buffer consisted of 50 mM KCl, 1 mM MgCl_2_, 50 mM Tris-HCl, pH 8.0, 2 mM ascorbic acid, 1 mM α-ketoglutarate and 100 μM (NH_4_)_2_Fe(SO_4_)_2_·6H_2_O. For demethylation assay of human ALKBH1, the same condition was applied except that MOPS buffer at pH 6.6 was used, which is adapted from previous report.^8^ Reactions were performed at 37 °C for 90 min, and stopped by adding EDTA to a final concentration of 5 mM (2.5 mM for reactions containing RNA). Then the products were subjected to dot blotting or digested for LC-MS/MS detection.

### Dot blotting

DNA samples were denatured at 95 °C for 10 min, snap-cooled on ice and neutralized with 10% volume of 6.6 M ammonium acetate. Samples (2 μL) were spotted on the membrane (Amersham Hybond-N+, GE), air-dried and then UV-cross-linked (2× auto-crosslink, 1800 UV Stratalinker, STRATAGENE). Membranes were blocked in blocking buffer (5% milk, PBS-T) for 2 h at room temperature, and incubated with *N*^6^-mA antibodies (202–003, Synaptic Systems, 1:1000) overnight at 4 °C. After 5-time washes, membranes were incubated with HRP-linked secondary anti-rabbit IgG antibody (ZB-2301, ZSGB-BIO, 1:5000) for 1 h at room temperature. Signals were detected with Immun-Star^TM^ WesternC^TM^ Chemiluminescence (Bio-Rad).

### LC-MS/MS detection of demethylation products as digested nucleosides

DNA was digested using DNA Degradase Plus (Zymo Research) by following the manufacturer’s instructions with small modification. In brief, the digestion was carried out at 37 °C for 70 min in a 40 μL volume containing 0.75 μM of oligos and 4 units of DNA Degradase Plus. As for RNA digestion, the oligos (1.5 μM) were first denatured at 95 °C for 5–10 min, snap-cooled on ice and then digested at 37 °C for 2 h by 2 units of Nuclease P1 (N8630, Sigma Aldrich) in a 30 μL volume containing 15 mM sodium acetate, pH 5.3 and 6 mM ZnCl_2_. Then the digestion was supplied with 250 mM NH_4_HCO_3_ and 0.6 unit of alkaline phosphatase (P4252, Sigma Aldrich), and incubated at 37 °C for an additional one hour. Following digestion, reaction mixture was centrifuged down at 10,000 × *g* for 10 min, and the supernatant was collected for LC-MS/MS analysis.

QExactive mass spectrometer (Thermo Fisher, CA), equipped with a heated electrospray ionization (HESI) probe was used in positive ion mode. Nucleosides were separated by a Luna omega PS C18 column (2.1 × 100 mm, 1.6 μm, Waters). A binary solvent system was used, in which mobile phase A consisted of 2 mM ammonium acetate and 100% aqueous, and mobile phase B of 100% acetonitrile. A 10-min gradient with flow rate of 200 μL/min was used as follows: 0–2 min at 1% B; 2–4 min, 1%–30% B; 4–6 min, 30%–98% B; 6–7.1 min, 98% B; 7.1–10 min, 1% B. Column chamber and sample tray were held at 35 °C and 10 °C, respectively. Data acquired in data-dependent MSMS acquisition mode. The MS and MSMS scans were collected with resolution of 70,000 and 17,500 respectively. The source parameters are as follows: spray voltage: 3000 V; ion transfer tube temperature: 320 °C; vaporizer temperature: 300 °C; sheath gas flow rate: 35 Arb; auxiliary gas flow rate: 10 Arb. Data analysis and quantitation were performed by the software Xcalibur 3.0.63 (Thermo Fisher, CA).

### LC-MS/MS detection of demethylation products as undigested DNA oligos

QEHF orbitrap coupled with Ultimate 3000 UHPLC (Thermo Fisher) was used for DNA analysis. BEH C18 column (1.0 × 50 mm, Waters) was applied in the analysis at flow rate of 0.15 mL/min. Mobile phase A consisted of 5 mM ammonium bicarbonate in aqueous solution. Mobile phase B contained 100% ACN. The gradient was as follows: 0 min, 2% B; 0.8 min, 2% B; 1.2 min, 98% B; 2.0 min, 98% B; 2.1 min, 2% B; 3.0 min, 2% B.

MS acquisition with mass ranges of m/z 1300–1900 was used in negative ion mode. Resolution of 120,000 was applied in the analysis. The detailed mass spectrometer parameters are as follows: spray voltage: 3.0 kV for negative; capillary temperature: 320 °C; heater temperature: 300 °C; sheath gas flow rate: 35; auxiliary gas flow rate: 10. Data was interpreted using Xcalibur (Thermo Fisher).

### Isothermal titration calorimetry

For ITC measurement, MnCl_2_, NOG and the proteins were prepared in the buffer containing 20 mM Tris-HCl, pH 7.5, and 100 mM NaCl. The titration was performed using a MicroCal iTC200 system (GE Healthcare) at 25 °C. Each ITC titration consisted of 17 successive injections with 0.4 μL for the first and 2.4 μL for the rest. MnCl_2_ at 1.0 mM was titrated into proteins at 0.1 mM; and NOG at 1.0 mM was titrated into proteins titrated with MnCl_2_, successively. The resultant ITC curves were processed using Origin 7.0 software (OriginLab) according to the “One Set of Sites” fitting model.

### Circular dichroism spectroscopy

Purified proteins were diluted to 0.1 mg/mL in 1× PBS buffer. Circular dichroism spectra were recorded under room temperature using an Applied Photo-physics Chirascan plus spectropolarimeter with a 1 mm path-length cell and a bandwidth of 1 nm. Spectra were scanned from 190–260 nm with a step size of 1 nm and were repeated for three times. Each reported circular dichroism curve was averaged with three scans after the subtraction of buffer control and smoothed.

### Electrophoretic mobility shift assay

The DNA substrate (41b6, 1 μM) was incubated with increasing amount of ALKBH1 proteins (0, 1, 5, 10, 15 μM) in buffer containing 20 mM Tris-HCl, pH 7.5, 100 mM NaCl, 0.1 mM MnCl_2_, and 1 mM NOG at 4 °C for 30 min. The samples were subjected to 5% native polyacrylamide gel and run at 100 V in 0.5× TBE buffer.

### Immunofluorescence

The stable ES cells carrying pLV-Flag-HA-Alkbh1 and pLV-Alkbh1-HA constructs were grown on gelatin-treated slides (Thermo, PEZGS0816) for 24 h. Cells were fixed with 1% PFA and permeabilized with 0.2% triton X-100 in 1 × phosphate buffered saline (PBS). Then the cells were incubated in the blocking buffer (2% BSA, 0.2% triton X-100 in PBS) for 1 h, incubated with 1:500 HA antibody (Cell signaling, 3724 S) in blocking buffer at 4 °C overnight, and then incubated with the secondary antibody (Thermo, A11004). DNA was stained with DAPI for 5 min. Slides were mounted with mounting gel (Electron Microscopy Sciences). Images were acquired with Leica SP5 confocal laser microscope.

### Single-stranded DNA-sequencing (ssDNA-seq)

ssDNA-seq was performed as the previously described protocol with minor modifications.^25^ ES cells (8 × 10^7^) cultured in feeder-free conditions on gelatin-coated tissue culture plates were washed with 1× PBS buffer at 37 °C. Low salt buffer (15 mM Tris-HCl, pH 7.5, 60 mM KCl, 15 mM NaCl, 5 mM MgCl_2_, 0.5 mM EGTA, and 300 mM sucrose) at 37 °C was added for 5 min. Cells were then treated with 100 mM KMnO_4_ for 80 s at 37 °C (“Treated” sample), and the reaction was quenched by the addition of 50 mM EDTA, 700 mM β-mercaptoethanol, and 1% (w/v) SDS. In parallel, the same number of cells were treated with water (“Blank” sample) and processed similarly. Lysates were incubated with 200 μg/mL of proteinase K (Qiagen, 19133) overnight at 37 °C. DNA was extracted twice with phenol. DNA was extracted a final time with phenol:chloroform:isoamyl alcohol (25:24:1, v/v/v; PCI), precipitated with 2 M ammonium acetate in ethanol, and washed with 70% ethanol (hereafter called PCI extraction with ethanol precipitation) and resuspended in 1 mL of 10 mM Tris-HCl, pH 8.0, and 1 mM EDTA buffer (TE buffer).

Genomic DNA was treated with an RNase A/T1 mix (Thermo EN0551; 1:50 final concentration, 40 μg/mL and 100 U/mL, respectively) for 1 h at 37 °C, then PCI extracted with ethanol precipitation and resuspended in 1 mL of TE buffer. Free 3′ ends formed due to DNA breakage during sample preparation were blocked by treatment with 100 μM cordycepin-5′-triphosphate sodium salt (Sigma Aldrich, C9137) and 400 U of Terminal transferase (NEB, M0315; TdT) in 1× TdT buffer in a reaction volume of 3 mL for 2 h at 37 °C. DNA was PCI extracted with ethanol precipitation and resuspended in 1 mL TE buffer.

Digestion of ssDNA was carried out by dividing each of the Treated and Blank samples equally into four micro-tubes with 0, 50, 100, or 200 U of S1 nuclease (ThermoFisher, EN0321) in 500 μL of the supplied reaction buffer and incubating for 30 min at 37 °C. DNA was PCI extracted with ethanol precipitation and resuspended in 100 μL TE buffer. Based on a fragment size distribution between 2–10 kb, the 50 U S1-treated samples (Treated and Blank) were chosen for further processing. Next, 35 μg of DNA was biotinylated with 300 U TdT, 250 μM dATP, 250 μM dCTP, and 50 μM Biotin-16-dUTP (Roche, 11093070910) in a final volume of 300 μL of 1× TdT buffer at 37 °C for 30 min. Reactions were stopped with 15 μL of 0.5 M EDTA. PCI extraction with ethanol precipitation was performed, followed by a second ethanol precipitation to remove free biotin, and samples were resuspended in 100 μL TE buffer.

Samples were sonicated with a Covaris S220 to generate DNA fragments between 200 and 700 bp and PCI extracted with ethanol precipitation to remove free biotin. DNA was resuspended in TE buffer, and 10% of the Treated sample was saved for the input control. With the remaining sample, biotinylated fragments were pulled down using streptavidin-coated beads using the manufacturer’s protocol (Dynabeads kilobaseBINDER Kit, ThermoFisher, 60101). After 2 additional washes in TE buffer, fragments were released by incubating beads with 50 U S1 nuclease in 100 μL reaction buffer for 15 min at 37 °C. Finally, DNA was purified with a MinElute PCR Purification Kit (Qiagen) and eluted in 30 μL of the manufacturer’s elution buffer. Sample concentrations were measured using a Qubit 3.0 fluorometer (Thermo Fisher Scientific), and at least a 10-fold greater pulldown efficiency for Treated vs. Blank was confirmed.

### Library preparation for high-throughput sequencing

“Treated” samples and their input controls were further processed for high-throughput sequencing. Sequencing libraries were made using the NEBNext Ultra II DNA Library Prep kit with 10–100 ng of each sample and input. Libraries were prepared according to the manufacturer’s instructions without size selection. Library concentrations were measured with Qubit and quality control performed with Agilent 2100 Bioanalyzer. Libraries were pooled and sequenced by paired-end 2 × 100 bp in one lane of an Illumina HiSeq4000.

### High-throughput sequencing data processing and analysis

Sequencing reads were filtered and pre-analyzed with the Illumina standard workflow. After filtering, high quality raw reads (in fastq format) were aligned to the mouse genome (UCSC, mm9) with Bowtie2 (2.2.9),^63^ allowing up to one mismatch in seed alignment. PCR and optical duplicates were removed with Picard (2.9.0; http://broadinstitute.github.io/picard). Tracks were generated using 10 bp bins, smoothed across 30 bp, and normalized to counts per million (CPM). After alignment and processing, ssDNA enriched regions were called with SICER (version 1.1, FDR < 0.01, input DNA as control),^64^ with window size 100 bp and gap size 200 bp. TT2 sample regions were called by only considering reads that had mapping quality scores of at least 20.

### Quantification of ssDNA enrichment in transposable elements

Raw reads in fastq format were analyzed by SalmonTE (version 0.8.2) with default parameters and fold enrichment of samples over input was calculated for each family of transposable elements in the mouse index.^65^ LINE1 subfamilies were assigned to bins of young, middle, and old age as performed previously.^6^

### Mouse ES cell culture

Mouse tetracycline-off *Cdx2* ES cells (C-ES)^66^ and TT2 ES cells were cultured on gelatin-coated tissue culture plates with recombinant LIF. ES cells were grown in DMEM supplemented with 15% fetal bovine serum, 1% non-essential amino acids, 1 mM sodium pyruvate, 2 mM l-glutamine, 1,000 units of mLIF (EMD Millipore), 0.1 mM β-mercaptoethanol (Sigma Aldrich) and antibiotics. Additionally, media for C-ES cells was supplemented with 0.5 μg/mL doxycycline to turn off *Cdx2* expression. Cells were passaged at 70%–80% confluence using 0.05% trypsin-EDTA.

### Antibodies and reagents for western blot and immunofluorescence

HA-Tag Rabbit mAb; CST; 3724; Clone C29F4; Lot 8; IF/WB; 1:1500/1:1000

Calnexin Antibody (H-70); SCBT; sc-11397; WB; 1:10,000

Mouse anti-α-Tubulin antibody; Sigma-Aldrich; T6074; Clone B-5-1-2; WB; 1:10,000

Donkey Anti-Rabbit IgG H&L Alexa Fluor^®^ 488; Abcam; ab150073; IF; 1:1000

Anti-rabbit IgG, HRP-linked Antibody; CST; 7074; Lot 28; WB; 1:6000

Anti-mouse IgG, HRP-linked Antibody; CST; 7076; Lot 33; Dot blot; 1:4000

Phalloidin-Tetramethylrhodamine B isothiocyanate; Sigma Aldrich; P1951; IF; 50 μg/mL.

### Data availability

The atomic coordinates and structure factors of mouse ALKBH1_37–369_, ALKBH1_1–359_ bound to Mn^2+^/NOG and ALKBH1_20–355_-DNA complex have been deposited in Protein Data Bank under accession codes 6IMA, 6IMC, and 6KSF, respectively.

## Supplementary information

Supplementary Figure S1

Supplementary Figure S2

Supplementary Figure S3

Supplementary Figure S4

Supplementary Figure S5

Supplementary Figure S6

Supplementary Figure S7

Supplementary Figure S8

Supplementary Figure S9

Supplementary Table S1

Supplementary Table S2

## References

[1] Koonin LAAEV (2001). The DNA-repair protein AlkB, EGL-9, and leprecan define new families of 2-oxoglutarate- and iron-dependent dioxygenases. Genome Biol..

[2] Kurowski MA, Bhagwat AS, Papaj G, Bujnicki JM (2003). Phylogenomic identification of five new human homologs of the DNA repair enzyme AlkB. BMC Genom..

[3] Johansson C (2014). The roles of Jumonji-type oxygenases in human disease. Epigenomics.

[4] Wei YF, Carter KC, Wang R-P, Shell BK (1996). Molecular cloning and functional analysis of a human cDNA encoding an Escherichia coli AlkB homolog, a protein involved in DNA alkylation damage repair. Nucleic Acids Res..

[5] Westbye MP (2008). Human AlkB homolog 1 is a mitochondrial protein that demethylates 3-methylcytosine in DNA and RNA. J. Biol. Chem..

[6] Wu TP (2016). DNA methylation on N(6)-adenine in mammalian embryonic stem cells. Nature.

[7] Haag S (2016). NSUN3 and ABH1 modify the wobble position of mt-tRNAMet to expand codon recognition in mitochondrial translation. EMBO J..

[8] Liu F (2016). ALKBH1-mediated tRNA demethylation regulates translation. Cell.

[9] Ougland R (2012). ALKBH1 is a histone H2A dioxygenase involved in neural differentiation. Stem Cells.

[10] Muller TA, Meek K, Hausinger RP (2010). Human AlkB homologue 1 (ABH1) exhibits DNA lyase activity at abasic sites. DNA Repair.

[11] Pan Z (2008). Impaired placental trophoblast lineage differentiation in Alkbh1(-/-) mice. Dev. Dyn..

[12] Nordstrand LM (2010). Mice lacking Alkbh1 display sex-ratio distortion and unilateral eye defects. PLoS ONE.

[13] Muller TA, Yu K, Hausinger RP, Meek K (2013). ALKBH1 is dispensable for abasic site cleavage during base excision repair and class switch recombination. PLoS ONE.

[14] Koziol MJ (2016). Identification of methylated deoxyadenosines in vertebrates reveals diversity in DNA modifications. Nat. Struct. Mol. Biol..

[15] Liu J (2016). Abundant DNA 6mA methylation during early embryogenesis of zebrafish and pig. Nat. Commun..

[16] Xiao C-L (2018). N6-methyladenine DNA modification in the human genome. Mol. Cell.

[17] Xie Q (2018). N6-methyladenine DNA modification in glioblastoma. Cell.

[18] Zhu S (2018). Mapping and characterizing N6-methyladenine in eukaryotic genomes using single-molecule real-time sequencing. Genome Res..

[19] Fu Y (2015). N6-methyldeoxyadenosine marks active transcription start sites in Chlamydomonas. Cell.

[20] Greer EL (2015). DNA methylation on N6-adenine in *C. elegans*. Cell.

[21] Zhang G (2015). N6-methyladenine DNA modification in *Drosophila*. Cell.

[22] Kigar SL (2017). N(6)-methyladenine is an epigenetic marker of mammalian early life stress. Sci. Rep..

[23] Yao B (2017). DNA N6-methyladenine is dynamically regulated in the mouse brain following environmental stress. Nat. Commun..

[24] Zhou C, Liu Y, Li X, Zou J, Zou S (2016). DNA N6-methyladenine demethylase ALKBH1 enhances osteogenic differentiation of human MSCs. Bone Res..

[25] Kouzine F (2017). Permanganate/S1 nuclease footprinting reveals non-B DNA structures with regulatory potential across a mammalian genome. Cell Syst..

[26] Zhao W (2017). BRCA1-BARD1 promotes RAD51-mediated homologous DNA pairing. Nature.

[27] Koh CWQ (2018). Single-nucleotide-resolution sequencing of human N6-methyldeoxyadenosine reveals strand-asymmetric clusters associated with SSBP1 on the mitochondrial genome. Nucleic Acids Res..

[28] Zhou C, Liu Y, Li X, Zou J, Zou S (2016). DNA N(6)-methyladenine demethylase ALKBH1 enhances osteogenic differentiation of human MSCs. Bone Res..

[29] Fu Y (2013). FTO-mediated formation of N6-hydroxymethyladenosine and N6-formyladenosine in mammalian RNA. Nat. Commun..

[30] Xiong J (2019). N6-Hydroxymethyladenine: a hydroxylation derivative of N6-methyladenine in genomic DNA of mammals. Nucleic Acids Res..

[31] Yu B (2006). Crystal structures of catalytic complexes of the oxidative DNA/RNA repair enzyme AlkB. Nature.

[32] Yang CG (2008). Crystal structures of DNA/RNA repair enzymes AlkB and ABH2 bound to dsDNA. Nature.

[33] Han Z (2010). Crystal structure of the FTO protein reveals basis for its substrate specificity. Nature.

[34] Aik W (2014). Structure of human RNA N6-methyladenine demethylase ALKBH5 provides insights into its mechanisms of nucleic acid recognition and demethylation. Nucleic Acids Res..

[35] Feng C (2014). Crystal structures of the human RNA demethylase Alkbh5 reveal basis for substrate recognition. J. Biol. Chem..

[36] Pastore C (2012). Crystal structure and RNA binding properties of the RNA recognition motif (RRM) and AlkB domains in human AlkB homolog 8 (ABH8), an enzyme catalyzing tRNA hypermodification. J. Biol. Chem..

[37] Sundheim O (2006). Human ABH3 structure and key residues for oxidative demethylation to reverse DNA/RNA damage. EMBO J..

[38] Wang G (2014). The atomic resolution structure of human AlkB homolog 7 (ALKBH7), a key protein for programmed necrosis and fat metabolism. J. Biol. Chem..

[39] Xu C (2014). Structures of human ALKBH5 demethylase reveal a unique binding mode for specific single-stranded N6-methyladenosine RNA demethylation. J. Biol. Chem..

[40] Sundheim O, Talstad VA, Vagbo CB, Slupphaug G, Krokan HE (2008). AlkB demethylases flip out in different ways. DNA Repair.

[41] Zhang X (2019). Structural insights into FTO’s catalytic mechanism for the demethylation of multiple RNA substrates. Proc. Natl Acad. Sci. USA.

[42] Holland PJ, Hollis T (2010). Structural and mutational analysis of Escherichia coli AlkB provides insight into substrate specificity and DNA damage searching. PLoS ONE.

[43] Hunt BYAJF (2009). Enzymological and structural studies of the mechanism of promiscuous substrate recognition by the oxidative DNA repair enzyme AlkB. PNAS.

[44] Yi C (2010). Iron-catalysed oxidation intermediates captured in a DNA repair dioxygenase. Nature.

[45] Bode J (2006). Correlations between scaffold/matrix attachment region (S/MAR) binding activity and DNA duplex destabilization energy. J. Mol. Biol..

[46] Michaël Weber HH (2003). Genomic imprinting controls matrix attachment regions in the Igf2 gene. Mol. Cell. Biol..

[47] Falnes, P. Ø., Johansen, R. F. & Seeberg, E. AlkB-mediated oxidative demethylation reverses DNA damage in Escherichia coli. *Nature* **419**, 178–182 (2002). 10.1038/nature01048 12226668

[48] Trewick SC, Henshaw TF, Hausinger RP, Lindahl T, Sedgwick B (2002). Oxidative demethylation by Escherichia coli AlkB directly reverts DNA base damage. Nature.

[49] Per Arne Aas MO (2003). Human and bacterial oxidative demethylases repair alkylation damage in both RNA and DNA. Nature.

[50] Shen L, Song CX, He C, Zhang Y (2014). Mechanism and function of oxidative reversal of DNA and RNA methylation. Annu. Rev. Biochem..

[51] Hu L (2013). Crystal structure of TET2-DNA complex: insight into TET-mediated 5mC oxidation. Cell.

[52] Zhao J, Bacolla A, Wagn G, Vasquez KM (2010). Non-B DNA structure-induced genetic instability and evolution. Cell. Mol. Life Sci..

[53] Bacolla A, Wells RD (2004). Non-B DNA conformations, genomic rearrangements, and human disease. J. Biol. Chem..

[54] Ghosh RP (2019). Satb1 integrates DNA binding site geometry and torsional stress to differentially target nucleosome-dense regions. Nat. Commun..

[55] Terumi K-S, Kohwi Y (1990). Torsional stress stabilizes extended base unpairing in suppressor sites flanking immunoglobulin heavy chain enhancer. Biochemistry.

[56] J Bode YK (1992). Biological significance of unwinding capability of nuclear matrix-associating DNAs. Science.

[57] Corless S, Gilbert N (2016). Effects of DNA supercoiling on chromatin architecture. Biophys. Rev..

[58] MINOR ZOAW (1997). Processing of X-ray diffraction data collected in oscillation mode. Methods Enzymol..

[59] Adams Paul D., Afonine Pavel V., Bunkóczi Gábor, Chen Vincent B., Davis Ian W., Echols Nathaniel, Headd Jeffrey J., Hung Li-Wei, Kapral Gary J., Grosse-Kunstleve Ralf W., McCoy Airlie J., Moriarty Nigel W., Oeffner Robert, Read Randy J., Richardson David C., Richardson Jane S., Terwilliger Thomas C., Zwart Peter H. (2010). PHENIX: a comprehensive Python-based system for macromolecular structure solution. Acta Crystallographica Section D Biological Crystallography.

[60] Vagin A, Teplyakov A (2010). Molecular replacement with MOLREP. Acta Crystallogr D Biol. Crystallogr..

[61] Emsley P, Cowtan K (2004). Coot: model-building tools for molecular graphics. Acta Crystallogr. D Biol. Crystallogr..

[62] Laskowski RA (1993). PROCHECK: a program to check the stereochemicai quality of protein structures. J. Appl. Cryst..

[63] Langmead B, Salzberg SL (2012). Fast gapped-read alignment with Bowtie 2. Nat. Methods.

[64] Zang C (2009). A clustering approach for identification of enriched domains from histone modification ChIP-Seq data. Bioinformatics.

[65] Jeong H-H, Yalamanchili HK, Guo C, Shulman JM, Liu Z (2018). An ultra-fast and scalable quantification pipeline for transposable elements from next generation sequencing data. Pac. Symp. Biocomput..

[66] Nishiyama A (2009). Uncovering early response of gene regulatory networks in ESCs by systematic induction of transcription factors. Cell Stem Cell.

