# Supplementary material for: Mammalian ALKBH1 serves as an N6-mA demethylase of unpairing DNA
Source: Cell Res. 2020 Feb 12;30(3):197–210. doi: 10.1038/s41422-019-0237-5 (PMC7054317; doi:10.1038/s41422-019-0237-5)
Supplement: Supplementary file 1 — Supplementary Figure S1 [file 41422_2019_237_MOESM1_ESM.pdf]

Supplementary information, Fig. S1

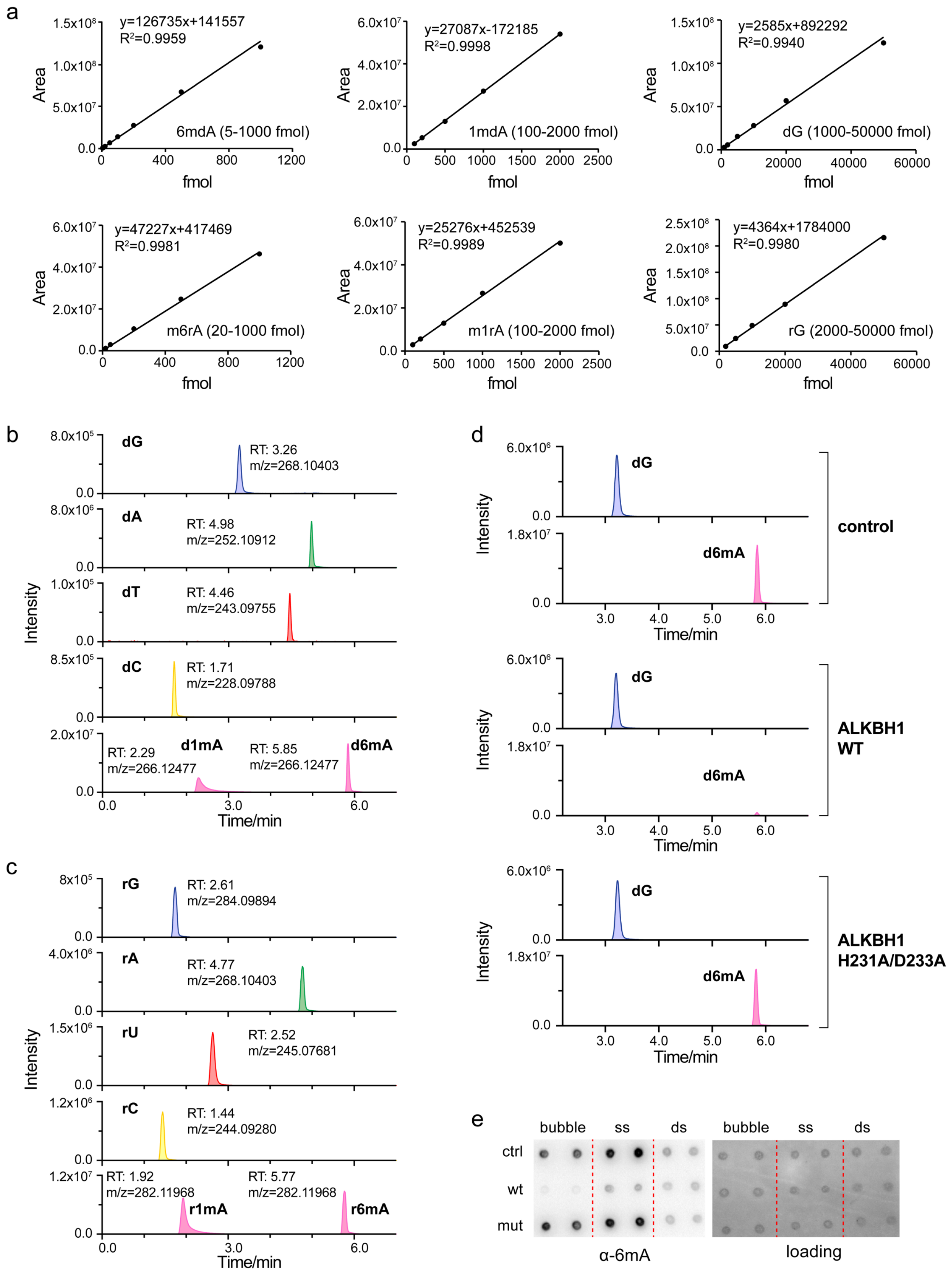

Supplementary information, Fig. S1|a, Standard curves for m6dA, m1dA, m6rA, m1rA, G and dG for quantification of digested nucleosides in UHPLC-Orbitrap MS. Linearity with R^2>0.99 was achieved for all nucleosides in the indicated ranges. **b-c**, Chromatograms of all deoxyribonucleoside/ribonucleoside and the methylated derivatives. **d**, Representatative MS spectra of dG and m6dA digested from bubbled DNA (41bp) after enzymatic assays using purified WT or mutant ALKBH1. DNA substrate from the reaction system without enzyme was used for negative control. The amount of dG and m6dA was quantified by the integrated area. **e**, Representative dot blot results of m6dA demethylation by ALKBH1 towards 41b6, 41ss, and 41ds.
