# Supplementary material for: Mammalian ALKBH1 serves as an N6-mA demethylase of unpairing DNA
Source: Cell Res. 2020 Feb 12;30(3):197–210. doi: 10.1038/s41422-019-0237-5 (PMC7054317; doi:10.1038/s41422-019-0237-5)
Supplement: Supplementary file 2 — Supplementary Figure S2 [file 41422_2019_237_MOESM2_ESM.pdf]

Supplymentary information, Fig. S2

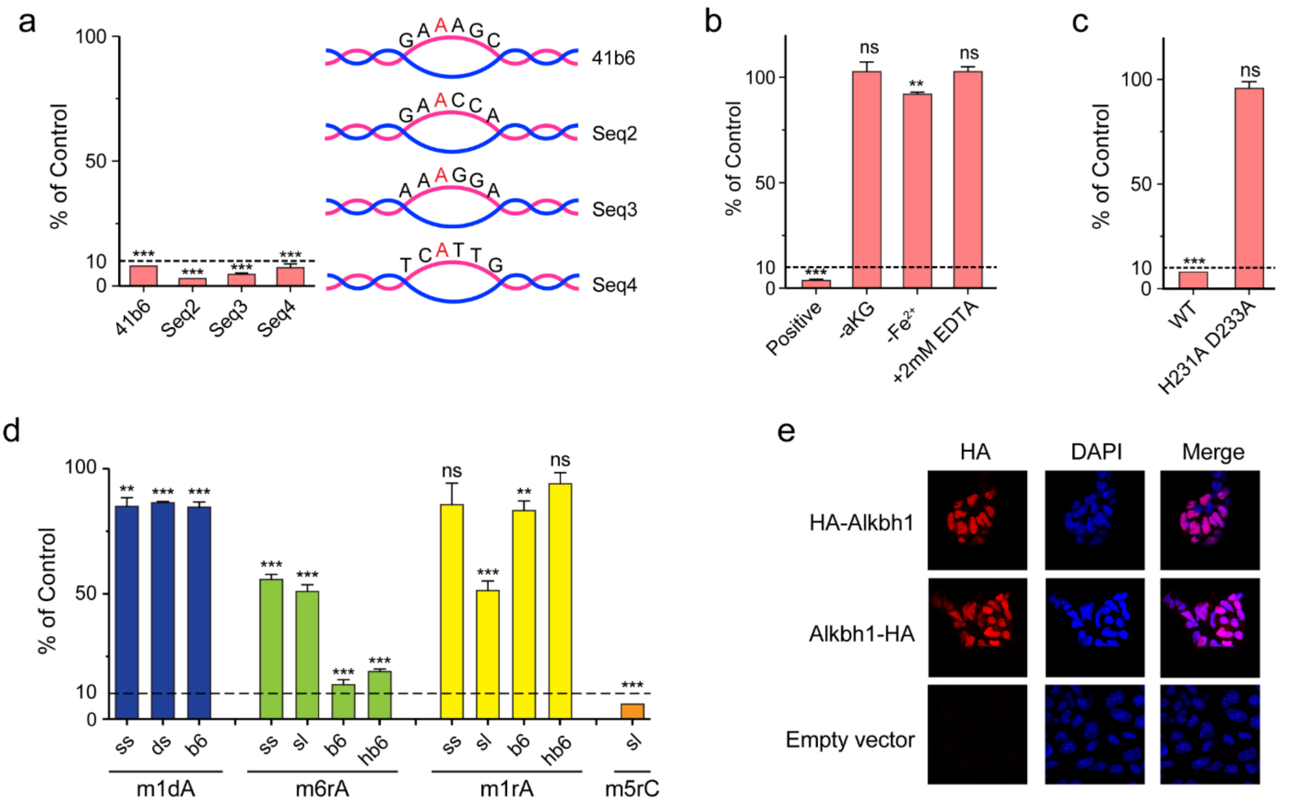

Supplymentary information, Fig. S2|a, *In vitro* N^6-mA demethylation using purified ALKBH1 and various bubbled sequences derived from SMART-seq data of mouse and human[1,2]. All substrates are 41bp in length with 6bp mismatched in the middle to generate bubbled DNA, and N^6-mAs are all located at position 3 as in 41b6. *** indicates P<0.001, *t*-test; error bars, ± s.d. of three biological replicates. **b**, Fe^{2+} and a-KG dependence of N^6-mA demethylation by ALKBH1. **c**, Loss of enzymatic activity of ALKBH1 after H231A/D233A double mutation. **d,** *In vitro* demethylation activities of ALKBH1 on m1dA, m6rA, m1rA, and m5rC. ss, ds, b6, and hb6 represent single-stranded DNA/RNA, double-stranded DNA/RNA, bubbled DNA-DNA/RNA-RNA hybrid (6bp mismatched as 41b6), and bubbled DNA-RNA hybrid with N^6-mA on DNA (6bp mismatched as 41b6), whose sequence are the same with that of 41b6. sl represents stemloop. sl of m1rA[3] and m5rC[4] are synthesized as previous publication, respectively. Sequence of sl of m6rA is the same with m1rA. ***, **, and ns indicate P<0.001, 0.01 and ≥0.05, respectively, *t*-test; error bars, ± s.d. of three biological replicates. **e**, Nuclear localization of ALKBH1 in mESc with transient overexpression of ALKBH1 tagged on N or C terminal.

Reference

1 Wu, T. P. *et al.* DNA methylation on N(6)-adenine in mammalian embryonic stem cells. *Nature* **532**, 329-333, doi:10.1038/nature17640 (2016).

2 Zhu, S. *et al.* Mapping and characterizing N6-methyladenine in eukaryotic genomes using single-molecule real-time sequencing. *Genome Res* **28**, 1067-1078, doi:10.1101/gr.231068.117 (2018).

3 Liu, F. *et al.* ALKBH1-Mediated tRNA Demethylation Regulates Translation. *Cell* **167**, 816-828 e816, doi:10.1016/j.cell.2016.09.038 (2016).

4 Haag, S. *et al.* NSUN3 and ABH1 modify the wobble position of mt-tRNAMet to expand codon recognition in mitochondrial translation. *EMBO J* **35**, 2104-2119, doi:10.15252/embj.201694885 (2016).
