# Supplementary material for: Mammalian ALKBH1 serves as an N6-mA demethylase of unpairing DNA
Source: Cell Res. 2020 Feb 12;30(3):197–210. doi: 10.1038/s41422-019-0237-5 (PMC7054317; doi:10.1038/s41422-019-0237-5)
Supplement: Supplementary file 3 — Supplementary Figure S3 [file 41422_2019_237_MOESM3_ESM.pdf]

Supplementary information, Fig. S3

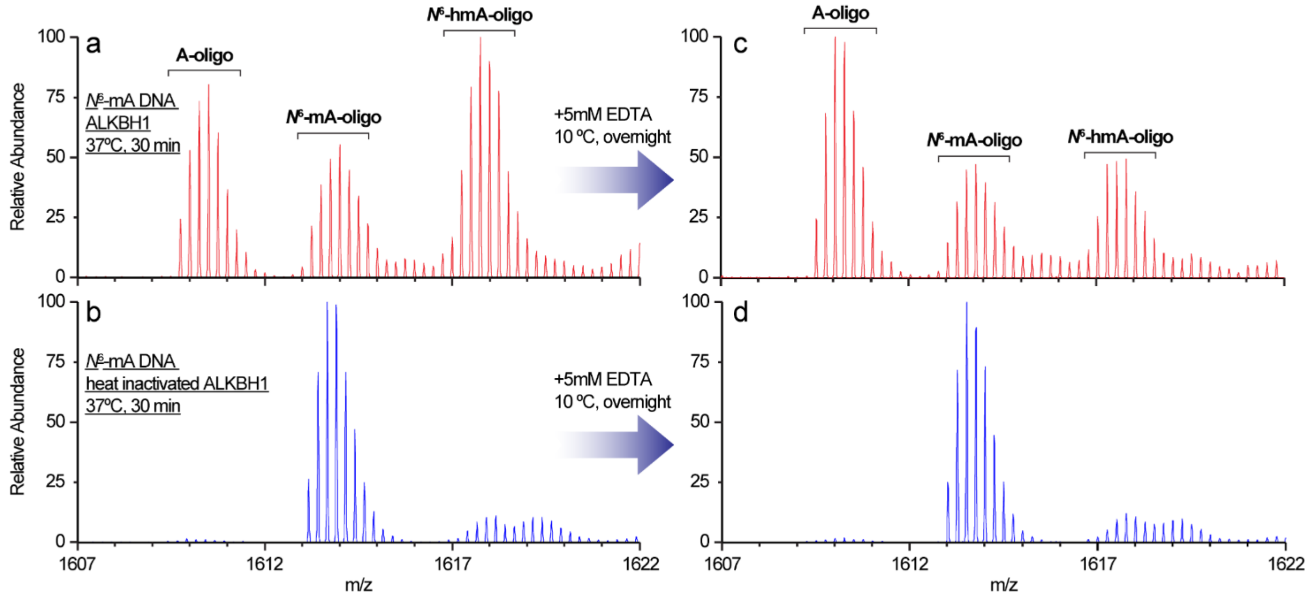

Supplementary information, Fig. S3| Decomposition of *N*^{6}-hmA analyzed as undigested oligoes. *In vitro* demethylation assay was set up as in Fig. 2c. An 18bp bulged DNA with one *N*^{6}-mA modification (1.5 μM) was treated by 1.5 μM of **a)** active and **b)** heat inactivated ALKBH1 for 30 minutes, and the reaction was stopped by 5 mM of EDTA. Samples were subjected to LC-MS/MS profiling **a)** and **b)** immediately, and **c)** and **d)** after overnight incubation at 10°C. Samples were analyzed as undigested oligoes by UHPLC-Orbitrap MS (see methods). All ions were detected adducts of $[M\text{-}4H]^{4-}$, and analyzed as in Fig. 2c. Monoisotopic peaks corresponding to *N*^{6}-hmA-oligo and dA-oligo appeared after treated by active ALKBH1 for 30 minutes. After reaction stop and overnight incubation at 10°C, *N*^{6}-hmA-oligo kept decreasing and dA-oligo kept increasing.
