# Supplementary material for: Mammalian ALKBH1 serves as an N6-mA demethylase of unpairing DNA
Source: Cell Res. 2020 Feb 12;30(3):197–210. doi: 10.1038/s41422-019-0237-5 (PMC7054317; doi:10.1038/s41422-019-0237-5)
Supplement: Supplementary file 4 — Supplementary Figure S4 [file 41422_2019_237_MOESM4_ESM.pdf]

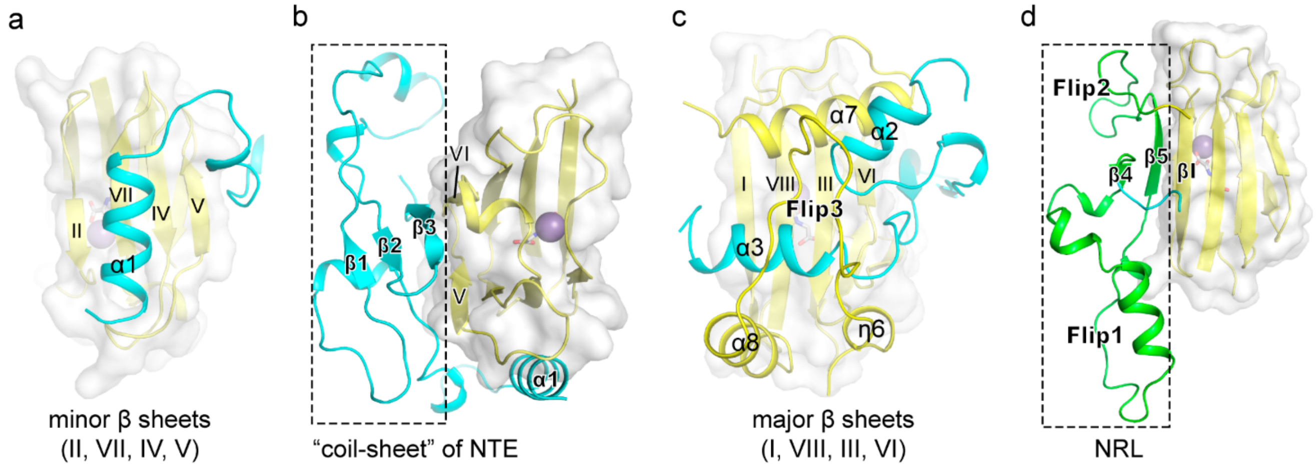

Supplementary information, Fig. S4| Stabilization of the central DSBH fold by structural elements from NTE and NRL. a, The "coil-sheet" structure of NTE extends the β(V-VI) edge from the back. **c**, A helical cluster stacks against the major sheet from the top. **d**, Flip1 and Flip2 extend the β(I) edge of the major sheet from the front. α1, Flip1 and Flip2, and the central jellyroll fold are colored in cyan, green, and yellow, respectively. Elements from NTE (cyan), NRL (green), and DSBH (yellow) are shown as ribbon. DSBH is also shown as surface for clarification. Mn(II) is shown as sphere (gray).
