# Supplementary material for: Mammalian ALKBH1 serves as an N6-mA demethylase of unpairing DNA
Source: Cell Res. 2020 Feb 12;30(3):197–210. doi: 10.1038/s41422-019-0237-5 (PMC7054317; doi:10.1038/s41422-019-0237-5)
Supplement: Supplementary file 6 — Supplementary Figure S6 [file 41422_2019_237_MOESM6_ESM.pdf]

Supplementary information, Fig. S6

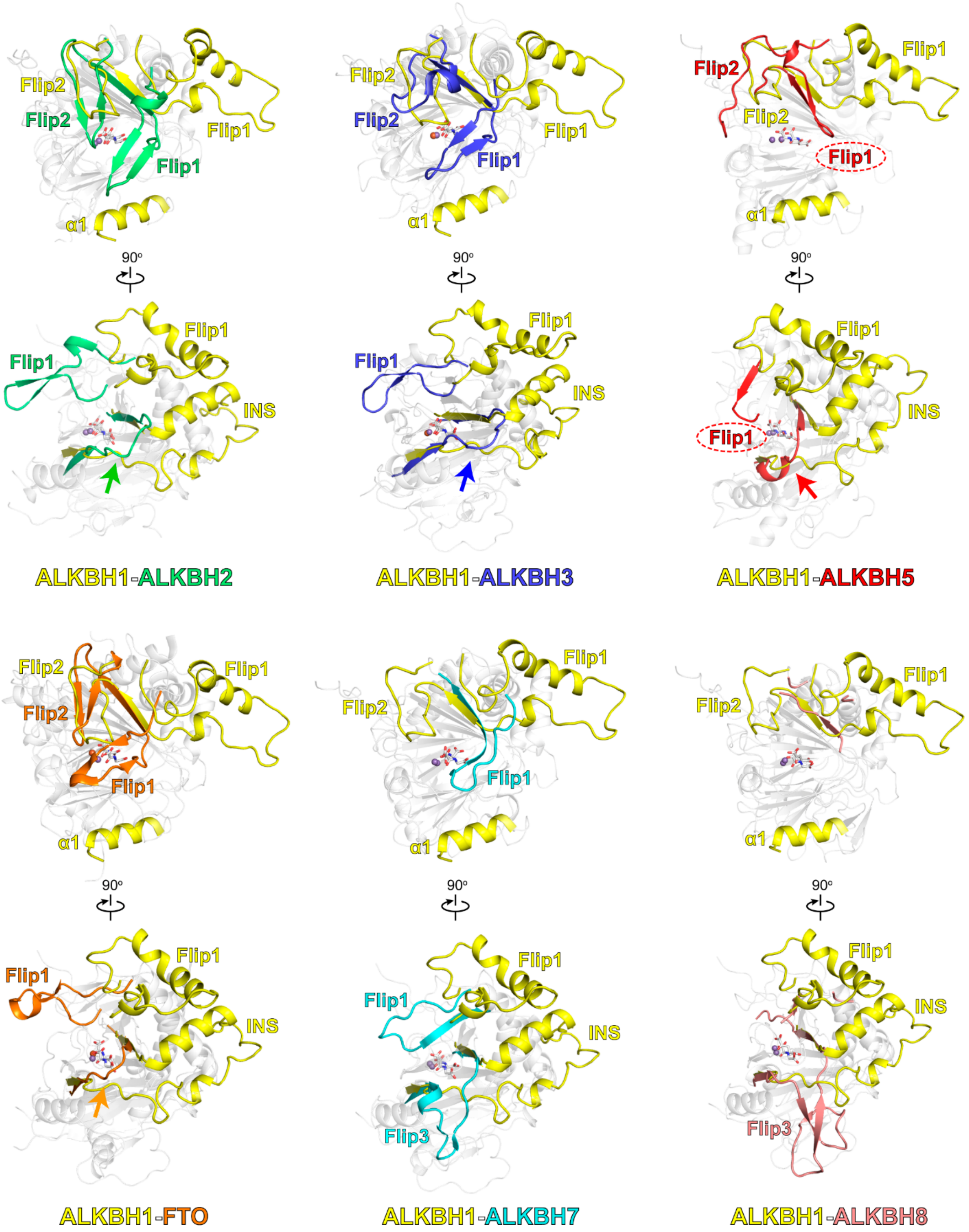

Supplementary information, Fig. S6| Structural comparison of Flip1 and ins between ALKBH1 and its paralogues in front and side views.
