# Supplementary material for: Mammalian ALKBH1 serves as an N6-mA demethylase of unpairing DNA
Source: Cell Res. 2020 Feb 12;30(3):197–210. doi: 10.1038/s41422-019-0237-5 (PMC7054317; doi:10.1038/s41422-019-0237-5)
Supplement: Supplementary file 7 — Supplementary Figure S7 [file 41422_2019_237_MOESM7_ESM.pdf]

## Supplementary information, Fig. S7

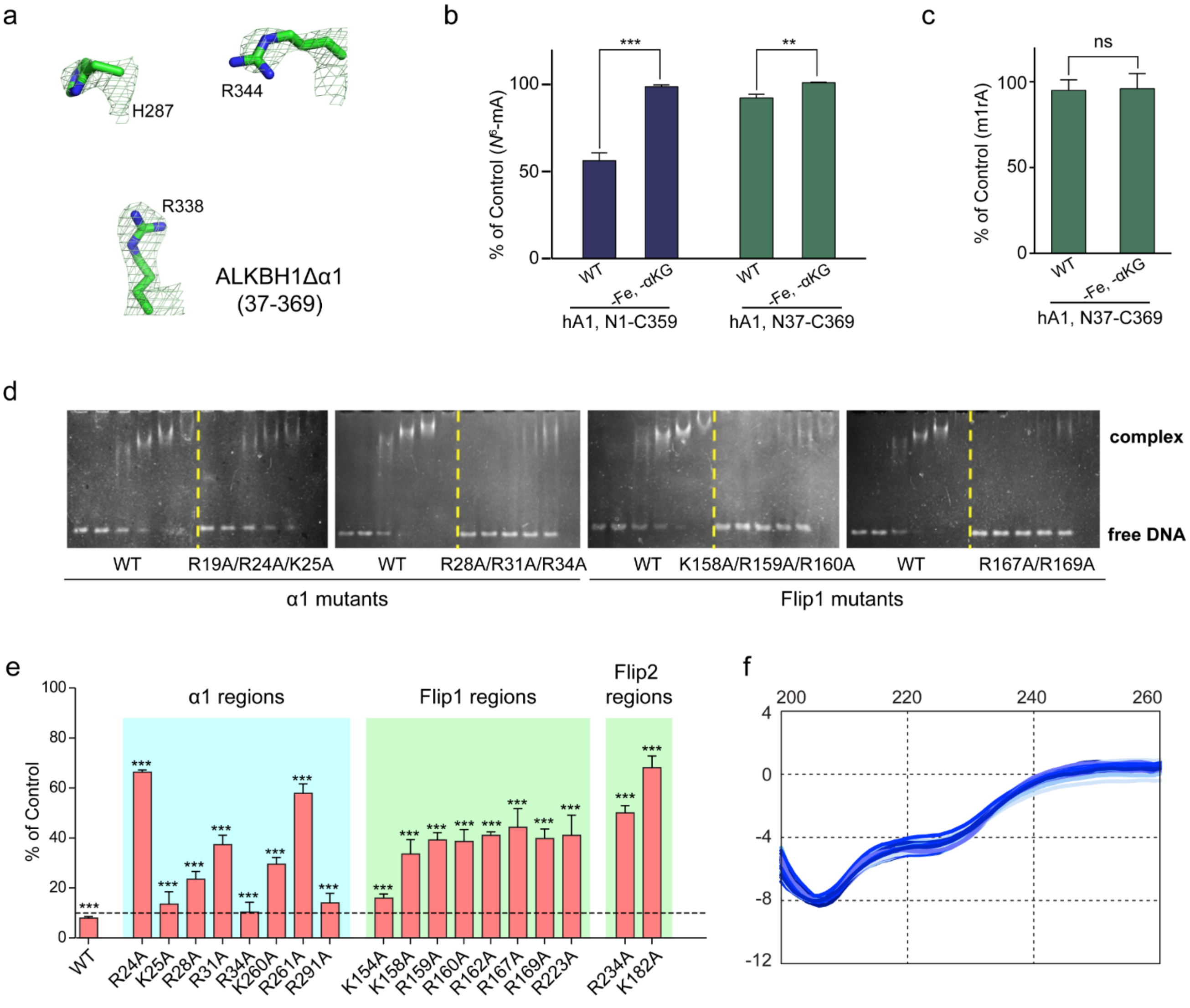


**Supplementary information, Fig. S7| a,** The close-up view of the catalytic center of ALKBH1Δα1. The 2$F_{obs}$-$F_{cal}$ omit maps for cofactor coordinating residues are shown. The maps were calculated at 1.8 Å and contoured at 1.4$\sigma$. **b,** *In vitro* demethylation activity towards $N^6$-mA in 41b6 DNA using human ALKBH1 with and without N-terminal α1 helix. **c,** *In vitro* demethylation activity towards 1rmA in stem loop (the same with 1rmA-sl in Extended Data Figure2) using human ALKBH1 without N-terminal α1 helix. **d**, Electrophoretic mobility shift assay. Multiple mutation of critical lysine and arginine (revealed by $N^6$-mA demethylation assay) on α1, Flip1 and Flip2 impairs the DNA binding affinity of ALKBH1. **e,** *In vitro* $N^6$-mA demethylation toward 41b6 catalyzed by wide type ALKBH1 and its mutants of annotated lysine or arginine on α1, Flip1 and Flip2. **f,** Circular dichroism curves comparing wide type ALKBH1 and its mutants.
