# Supplementary material for: Mammalian ALKBH1 serves as an N6-mA demethylase of unpairing DNA
Source: Cell Res. 2020 Feb 12;30(3):197–210. doi: 10.1038/s41422-019-0237-5 (PMC7054317; doi:10.1038/s41422-019-0237-5)
Supplement: Supplementary file 8 — Supplementary Figure S8 [file 41422_2019_237_MOESM8_ESM.pdf]

Supplementary information, Fig. S8

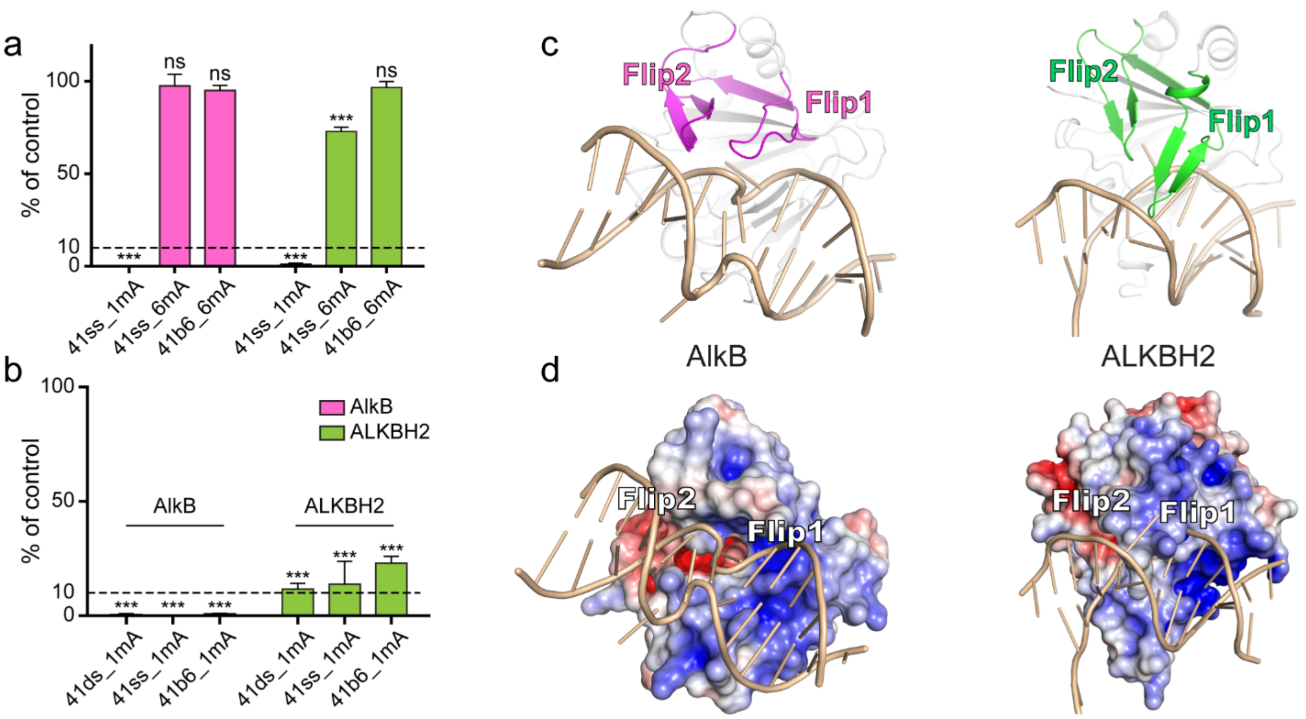

Supplementary information, Fig. S8| a, *In vitro* demethylation of 1mA in ssDNA (41ss_1mA) and N^6-mA catalyzed in ssDNA (41ss_6mA) and bubbled DNA (41b6_6mA) by AlkB and ALKBH2. **b,** *In vitro* demethylation of 1mA in dsDNA (41ds), ssDNA (41ss), and bubbled DNA (41b6) by AlkB and ALKBH2. **c**, Ribbon representation of AlkB-dsDNA (PDB: 3BI3) and ALKBH2-dsDNA (PDB: 3BUC) complex structure. The NRL subdomain (containing Flip1 and Flip2) of AlkB and ALKBH2 are colored in magenta and green, respectively, and the dsDNAs are colored in wheat. **d,** Electrostatic potential surface view of the two complex structures in c. *, **, ***, and ns indicate $P<0.05$, 0.01, 0.001 and ≥0.05, respectively, *t*-test; error bars, ± s.d. of three biological replicates.
