# Supplementary material for: Mammalian ALKBH1 serves as an N6-mA demethylase of unpairing DNA
Source: Cell Res. 2020 Feb 12;30(3):197–210. doi: 10.1038/s41422-019-0237-5 (PMC7054317; doi:10.1038/s41422-019-0237-5)
Supplement: Supplementary file 9 — Supplementary Figure S9 [file 41422_2019_237_MOESM9_ESM.pdf]

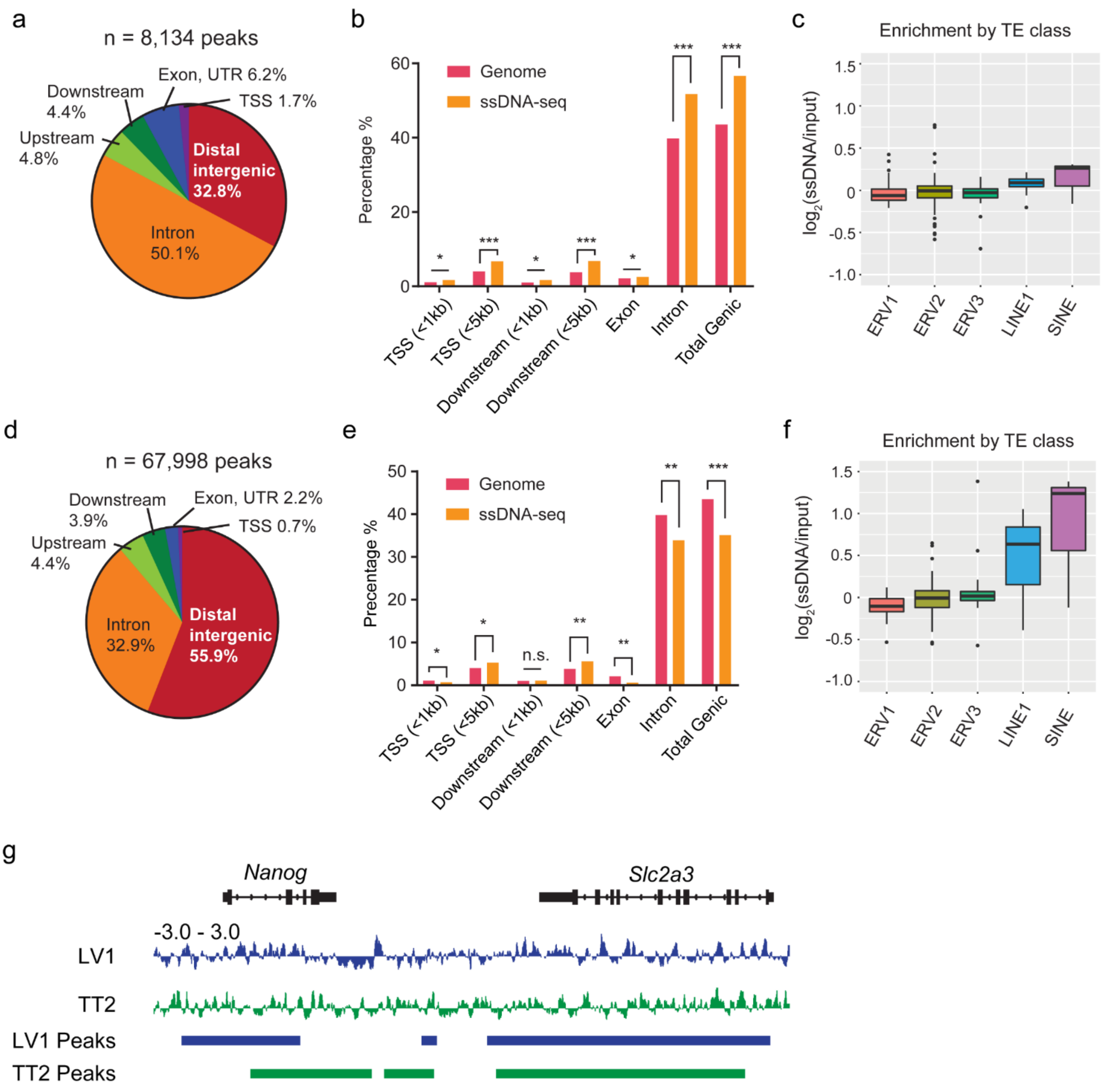

Supplementary information, Fig. S9| a, LV1 and **d,** TT2 cell line ssDNA-seq data peak annotation by CEAS (version 1.0.0); **b,** LV1 and **e,** TT2 enrichment of ssDNA-seq peaks compared to expected genomic distribution *$P < 10^{-20}$, **$P < 10^{-100}$, ***$P < 10^{-200}$; **c,** LV1 and **f,** TT2 read enrichment by transposable element class using SalmonTE; **g,** Representative track of LV1 and TT2 ssDNA-seq shown as $\log_2$(ssDNA/input) at the pluripotency gene *Nanog*.
