# Supplementary material for: Mammalian ALKBH1 serves as an N6-mA demethylase of unpairing DNA
Source: Cell Res. 2020 Feb 12;30(3):197–210. doi: 10.1038/s41422-019-0237-5 (PMC7054317; doi:10.1038/s41422-019-0237-5)
Supplement: Supplementary file 10 — Supplementary Table S1 [file 41422_2019_237_MOESM10_ESM.pdf]

Supplementary information, Table S1. Nucleic acid substrates

Name	Sequence
41_b6 (41_b6_pos3)	F: 5'-CATGATACCTTATGGA(6mA)AGCATGCTTGTATTTCTTATGAAC-3'<br>R: 5'-GTTCATAAGAAATACAAGCATTAGGTGCATAAGGTATCATG-3'
41_ds	F: 5'-CATGATACCTTATGGA(6mA)AGCATGCTTGTATTTCTTATGAAC-3'<br>R: 5'-GTTCATAAGAAATACAAGCATGCTTTCCATAAGGTATCATG-3'
41_ss	F: 5'-CATGATACCTTATGGA(6mA)AGCATGCTTGTATTTCTTATGAAC-3'
41_b16	F: 5'-CATGATACCTTATGGA(6mA)AGCATGCTTGTATTTCTTATGAAC-3'<br>R: 5'-GTTCATAAGAAATACATTGTTTAGGTGTTATTGGTATCATG-3'
41_b12	F: 5'-CATGATACCTTATGGA(6mA)AGCATGCTTGTATTTCTTATGAAC-3'<br>R: 5'-GTTCATAAGAAATACAAGGTTTAGGTGTTGAAGGTATCATG-3'
41_b9	F: 5'-CATGATACCTTATGGA(6mA)AGCATGCTTGTATTTCTTATGAAC-3'<br>R: 5'- GTTCATAAGAAATACAAGCAGTAGGTGTGTAAGGTATCATG -3'
41_b5 (41_b5_pos3)	F: 5'-CATGATACCTTATGGA(6mA)AGCATGCTTGTATTTCTTATGAAC-3'<br>R: 5'-GTTCATAAGAAATACAAGCATGAGGAGCATAAGGTATCATG-3'
41_b3 (bubble_3)	F: 5'-CATGATACCTTATGGA(6mA)AGCATGCTTGTATTTCTTATGAAC-3'<br>R: 5'- GTTCATAAGAAATACAAGCATGCGGACCATAAGGTATCATG-3'
41_b1	F: 5'-CATGATACCTTATGGA(6mA)AGCATGCTTGTATTTCTTATGAAC-3'<br>R: 5'-GTTCATAAGAAATACAAGCATGCTGTCCATAAGGTATCATG-3'
41_b6_pos1	F: 5'-TGATACCTTATGGA(6mA)AGCATGCTTGTATTTCTTATGAACCA-3'<br>R: 5'-TGGTTCATAAGAAATACAAGCTCTGAATCCATAAGGTATCA-3'
41_b6_pos2	F: 5'-ATGATACCTTATGGA(6mA)AGCATGCTTGTATTTCTTATGAACC-3'<br>R: 5'-GGTTCATAAGAAATACAAGCACTGAAACCATAAGGTATCAT-3'
41_b6_pos4	F: 5'-CCATGATACCTTATGGA(6mA)AGCATGCTTGTATTTCTTATGAA-3'<br>R: 5'-TTCATAAGAAATACAAGCATGGAAAGGATAAGGTATCATGG-3'
41_b6_pos5	F: 5'-ACCATGATACCTTATGGA(6mA)AGCATGCTTGTATTTCTTATGA-3'<br>R: 5'-TCATAAGAAATACAAGCATGCAAAGGTTAAGGTATCATGGT-3'
41_b6_pos6	F: 5'-ACCATGATACCTTATGGA(6mA)AGCATGCTTGTATTTCTTATGA-3'<br>R: 5'-TCATAAGAAATACAAGCATGCTAAGGTAAAGGTATCATGGT-3'
bulge3_A1	F: 5'-CATGATACCTTATGG(6mA)AACATGCTTGTATTTCTTATGAAC-3'<br>R: 5'-GTTCATAAGAAATACAAGCATGCCATAAGGTATCATG-3'
bulge3_A2	F: 5'-CATGATACCTTATGGA(6mA)AGCATGCTTGTATTTCTTATGAAC-3'<br>R: 5'-GTTCATAAGAAATACAAGCATGCCCATAAGGTATCATG-3'
bulge3_A3	F: 5'-CATGATACCTTATGAA(6mA)GCATGCTTGTATTTCTTATGAAC-3'<br>R: 5'-GTTCATAAGAAATACAAGCATGCCATAAGGTATCATG-3'
hairpin_1	5'-TCGGGCC(6mA)ATACCCCGA-3'
hairpin_2	5'-CCGATCACCTCCGATGCCGATAATCTCATCGGCATCGG(6mA)GAAAGCATA-3'
hairpin_3	5'-CGATCACATCCGATGCCGATAATCTCATCGGCATCGG(6mA)GAAAGCAT-3'
hairpin_4	5'-CGATCACAGCCGATGCCGATAATCTCATCGGCATCGG(6mA)GAAAGCAT-3'
cruciform	F: 5'-AATCTACTTTCTTTG(6mA)GAAACTAGCAATCCCATTTGGATTGCTAGTTTCGCTTTTATATCAATAA-3'<br>R: 5'-TTATTGATATAAAAGAGAAACTAGCAATCCAAATGGGATTGCTAGTTTCGCAAAGAAAGTAGATT-3'
replication folk	8: 5'-GACGCTGCCGAATTCTACCAGTGCCTTGCT(6mA)GGACATCTTTGCCCACCTGCAGGTTCACCC-3'<br>9: 5'-GGACATCTTTGCCCACCTGCAGGTTCACCC-3'<br>10: 5'-GGGTGAACCTGCAGGTGGGCAAAGATGTCC-3'<br>11: 5'-GGGTGAACCTGCAGGTGGGCAAAGATGTCCCAGCAAGGCACTGGTAGAATTCGGCAGCGTC-3'
R_loop	F: 5'-TTCTTATGAACCATGATACCTTATGGA(6mA)AGCATGCTTGTATTTCTTATGAACCAT-3'<br>R: 5'-ATGGTTCATAAGAAATACAAGGTTTAGGTGTTGAAGGTATCATGGTTCATAAGAA-3'<br>RNA_12: 5'-CAACACCUAAAC-3'

D_loop	F: 5’-TTCTTATGAACCATGATACCTTATGGA(6mA)AGCATGCTTGTATTTCTTATGAACCAT-3’<br>R: 5’-ATGGTTCATAAGAAATACAAGGTTTAGGTGTTGAAGGTATCATGGTTCATAAGAA-3’<br>DNA_12: 5’-CAACACCTAAAC-3’
Seq2 (41bp, bubble6)	F: 5’-GCTCAGTACGAGAGGA(6mA)CCAAAGGTTCAGACATTTGGTGTA-3’<br>R: 5’-TACACCAAATGTCTGAACCTTACCAAACTCTCGTACTGAGC-3’
Seq3 (41bp, bubble6)	F: 5’-ATTGAATGGAAATGAA(6mA)GGAGTCATCAAATAATGGAATCGC-3’<br>R: 5’-GCGATTCCATTATTTGATGACAGTAGACATTTCCATTCAAT-3’
Seq4 (41bp, bubble6)	F: 5’-GGAATCTAATGGAATC(6mA)TTGAAAGGAATTGAATGGAATCGT-3’<br>R: 5’-ACGATTCCATTCAATTCCTTTGTTACTTTCCATTAGATTCC-3’
41_b6 (1mA)	F: 5’-CATGATACCTTATGGA(1mA)AGCATGCTTGTATTTCTTATGAAC-3’<br>R: 5’-GTTCATAAGAAATACAAGCATTAGGTGCATAAGGTATCATG-3’
41_ds (1mA)	F: 5’-CATGATACCTTATGGA(1mA)AGCATGCTTGTATTTCTTATGAAC-3’<br>R: 5’-GTTCATAAGAAATACAAGCATGCTTTCCATAAGGTATCATG-3’
41_ss (1mA)	F: 5’-CATGATACCTTATGGA(1mA)AGCATGCTTGTATTTCTTATGAAC-3’
41_b6 (m6rA)	F: 5’-CAUGAUACCUUAUGGA(m6A)AGCAUGCUUGUAUUUCUUAUGAAC-3’<br>R: 5’-GUUCAUAAGAAAUACAAGCAUUAGGUGCAUAAGGUAUCAUG-3’
41_b6 (m6rA) Hybrid	F: 5’-CAUGAUACCUUAUGGA(m6A)AGCAUGCUUGUAUUUCUUAUGAAC-3’<br>R: 5’-GTTCATAAGAAATACAAGCATTAGGTGCATAAGGTATCATG-3’
41_ss (m6rA)	F: 5’-CAUGAUACCUUAUGGA(m6A)AGCAUGCUUGUAUUUCUUAUGAAC-3’
sl (m6rA)	F: 5’-CCCGGUUCG(m6A)UUCCCGG-3’
41_b6 (m1rA)	F: 5’-CAUGAUACCUUAUGGA(m1A)AGCAUGCUUGUAUUUCUUAUGAAC-3’<br>R: 5’-GUUCAUAAGAAAUACAAGCAUUAGGUGCAUAAGGUAUCAUG-3’
41_b6 (m1rA) Hybrid	F: 5’-CAUGAUACCUUAUGGA(m1A)AGCAUGCUUGUAUUUCUUAUGAAC-3’<br>R: 5’-GTTCATAAGAAATACAAGCATTAGGTGCATAAGGTATCATG-3’
41_ss (m1rA)	F: 5’-CAUGAUACCUUAUGGA(m1A)AGCAUGCUUGUAUUUCUUAUGAAC-3’
sl (m1rA)	F: 5’-CCCGGUUCG(m1A)UUCCCGG-3’
sl (m5rC)	F: 5’-UCGGGCC(5mrC)AUACCCCGA-3’
18_bulge3*	F: 5’-ACCTTATGGA(6mA)AGCATGCTTG-3’<br>R: 5’-CAAGCATGCCCATAAGGT
21_bulge3**	F: 5’-GCTGAGTGCC*CGCGTGCTGGATCC-3’<br>R: 5’- GGATCCAGCACGCCACTCAGC-3’

* 18_bulge3 DNA and its forward strand were used for kinetics studies (Fig. 6d).
** 21_bulge3 DNA was used for generation of ALKBH1-DNA crosslinked complex. C* denotes the disulfide modified cytosine for crosslinking.
