# Supplementary material for: Mammalian ALKBH1 serves as an N6-mA demethylase of unpairing DNA
Source: Cell Res. 2020 Feb 12;30(3):197–210. doi: 10.1038/s41422-019-0237-5 (PMC7054317; doi:10.1038/s41422-019-0237-5)
Supplement: Supplementary file 11 — Supplementary Table S2 [file 41422_2019_237_MOESM11_ESM.pdf]

Supplementary information, Table S2. Data collection and refinement statistics

**Crystal**	**ALKBH1$_{37-369}$**	**ALKBH1$_{1-359}$**	**ALKBH1$_{20-355}$-DNA**
**Data Collection**	SeMet-SAD[$]		
Beamline	SSRF-BL19U	SSRF-BL17U	SSRF-BL17U
Wavelength (Å)	0.9785	0.9792	0.9791
Space group	C2	I4	$P4_12_12$
Unit Cell			
a, b, c (Å)	203.1, 56.1, 105.7	147.9, 147.9, 178.0	67.5, 67.5, 263.6
α, β, γ (°)	90, 117.9, 90	90, 90, 90	90, 90, 90
Resolution (Å)	50-2.6 (2.64-2.60)*	50-2.5 (2.54-2.50)	50-2.4 (2.44-2.40)
R_{sym} (%)	8.8 (75.2)	9.0 (77.6)	4.7 (71.6)
CC1/2 (%)	98.8 (53.8)	99.9 (87.4)	100.0 (89.6)
$I/\sigma (I)$	11.3 (1.2)	16.5 (1.9)	26.7 (2.5)
Completeness (%)	98.6 (99.2)	99.8 (99.9)	98.8 (97.8)
Redundancy	3.0 (3.0)	6.0 (6.0)	8.2 (8.6)
**Refinement (F>0)**			
Resolution (Å)	44.3-2.6	35.2-2.5	32.9-2.4
No. of unique reflections	62,965/32,489[#]	64,478	24,541
R_{work}/R_{free} (%)	19.0/23.9	18.0/22.3	22.8/27.2
No. of non-H atoms			
Protein	6851	10,843	2672
DNA	-	-	912
Water	104	351	63
Mn^{2+}/NOG	–	4/40	
Average B-factors (Å^2)			
Protein	57.4	46.4	55.5
DNA	-	-	63.4
Water	44.6	43.7	48.3
R.m.s. deviations			
Bond lengths (Å)	0.003	0.008	0.003
Bond angles (°)	0.599	0.917	0.781
Ramachandran plot (%)			
Most favored	90.8	92.0	92.0
Additional allowed	9.1	7.9	7.3
Generously allowed	0.1	0.1	0.7

[$]Data collection and refinement statistics in this column are based on anomalous scaling

*Highest-resolution shell is shown in parentheses

[#]Non-anomalous reflections
